# A gene silencing screen uncovers diverse tools for targeted gene repression in *Arabidopsis*

**DOI:** 10.1038/s41477-023-01362-8

**Published:** 2023-03-06

**Authors:** Ming Wang, Zhenhui Zhong, Javier Gallego-Bartolomé, Zheng Li, Suhua Feng, Hsuan Yu Kuo, Ryan L. Kan, Hoiyan Lam, John Curtis Richey, Linli Tang, Jessica Zhou, Mukun Liu, Yasaman Jami-Alahmadi, James Wohlschlegel, Steven E. Jacobsen

**Affiliations:** 1 Department of Molecular, Cell and Developmental Biology, University of California at Los Angeles, Los Angeles, CA USA; 2 Eli & Edythe Broad Center of Regenerative Medicine & Stem Cell Research, University of California at Los Angeles, Los Angeles, CA USA; 3 Department of Statistics, University of California, Riverside, CA USA; 4 Department of Biological Chemistry, University of California at Los Angeles, Los Angeles, CA USA; 5 Howard Hughes Medical Institute (HHMI), University of California at Los Angeles, Los Angeles, CA USA; 6 Present Address: Instituto de Biología Molecular y Celular de Plantas (IBMCP), CSIC-Universitat Politècnica de València, Valencia, Spain

**Keywords:** Gene silencing, Molecular engineering in plants, Genetic engineering

## Abstract

DNA methylation has been utilized for target gene silencing in plants. However, it is not well understood whether other silencing pathways can be also used to manipulate gene expression. Here we performed a gain-of-function screen for proteins that could silence a target gene when fused to an artificial zinc finger. We uncovered many proteins that suppressed gene expression through DNA methylation, histone H3K27me3 deposition, H3K4me3 demethylation, histone deacetylation, inhibition of RNA polymerase II transcription elongation or Ser-5 dephosphorylation. These proteins also silenced many other genes with different efficacies, and a machine learning model could accurately predict the efficacy of each silencer on the basis of various chromatin features of the target loci. Furthermore, some proteins were also able to target gene silencing when used in a dCas9-SunTag system. These results provide a more comprehensive understanding of epigenetic regulatory pathways in plants and provide an armament of tools for targeted gene manipulation.

## Abstract (web summary)

Using a gain-of-function method, this work identified 14 plant proteins that can repress gene expression via diverse epigenetic pathways, including DNA methylation, histone modifications and interference of RNA polymerase II transcription.

## Main

Transcriptional gene regulation is a fundamental biological process that controls the on or off states of gene expression, and involves DNA methylation, histone modification and chromatin remodelling^1^. In plants, DNA methylation is generally linked to transcriptional gene silencing^2^. For example, the *Arabidopsis FWA* gene is normally DNA methylated and silenced in all tissues, except in the developing endosperm where it is demethylated and expressed^3^.

In addition to DNA methylation, histone modifications also contribute to gene silencing^1^. For example, polycomb repressive complex (PRC) 1 and PRC2 are conserved in plants and animals and act to silence genes via the histone mark H3K27 trimethylation (me3) (refs. ^4–6^). Gene silencing can be also achieved by removing activating histone marks. For example, histone H3K4me3 and histone acetylation are associated with active gene activity, which can be erased through H3K4 demethylases such as Jumonji-containing proteins (JMJs), and histone deacetylases (HDACs), respectively^7–9^.

Artificial zinc fingers are DNA binding domains that can be designed to bind a specific sequence and guide fusion proteins to specific loci^10^. For example, artificial zinc finger 108 (hereafter ZF) was designed to bind the *Arabidopsis* *FWA* promoter in the region that is normally methylated in Col-0 wild-type plants^11^. When ZF was fused with the RNA-directed DNA methylation (RdDM) component SUVH9 and transformed into *fwa* epiallele-containing plants, *FWA* DNA methylation and suppression were restored^11^. It was later shown that ZF fusions with many other RdDM-related proteins also caused *FWA* silencing and methylation^12–14^. However, it is largely unknown whether ZF fusions with non-DNA-methylation-related chromatin proteins can also trigger target gene silencing^15^.

In this Article, we fused a panel of 270 putative *Arabidopsis* chromatin proteins to ZF, and screened for fusions capable of silencing *FWA*. We identified 14 proteins capable of silencing through diverse mechanisms including establishment of DNA methylation, H3K27me3 deposition, H3K4me3 demethylation, H3K9, H3K14, H3K27 and H4K16 deacetylation, inhibition of RNA polymerase II (Pol II) transcriptional elongation, or Pol II dephosphorylation. We found that some target genes were only silenced by certain effector proteins, and a machine learning model could accurately predict which genes would be effectively silenced based on proximal chromatin features and expression levels of the target genes. Some proteins were also able to target gene silencing using the CRISPR/dCas9 based SunTag system^14,16^. These findings lay a foundation for more detailed mechanistic understanding of gene silencing pathways and provide an array of new tools for targeted gene silencing.

## Results

### A gain-of-function screen for regulators of gene silencing

We utilized the native *Arabidopsis* gene *FWA* as a reporter to screen for regulators of gene silencing. *FWA* encodes a transcription factor that causes a late flowering phenotype when overexpressed, resulting in a greater number of leaves produced before flowering. In Col-0 wild-type plants, *FWA* is completely silenced by DNA methylation, while in *fwa* epialleles that have permanently lost this DNA methylation, *FWA* misexpression causes late flowering^17^. To find putative gene silencing regulators, we searched the *Arabidopsis* ORFeome collections^18,19^ for chromatin-related proteins and also added other proteins of interest that were not present in the ORFeome collections (Supplementary Table 1). A total of 270 putative *Arabidopsis* chromatin proteins were fused with a ZF designed to bind the *FWA* promoter^11^. These fusions were individually transformed into *fwa* plants to screen for regulators that triggered *FWA* silencing and restored an early flowering phenotype.

**Fig. 1:**
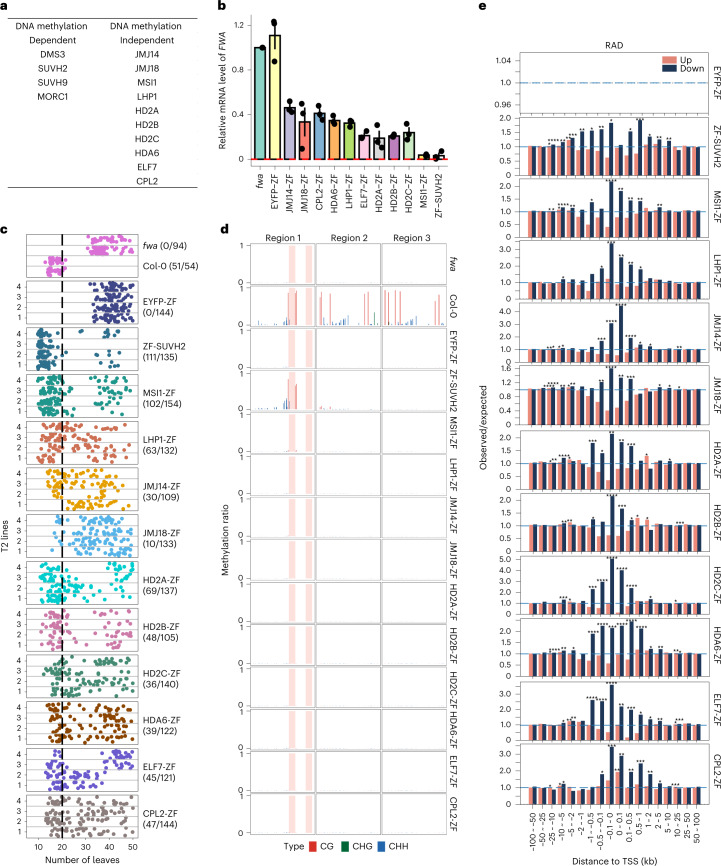
The effector proteins obtained from ZF target screening. **a**, The list of effector proteins identified from ZF target screening, which were dependent (left) or independent (right) of DNA methylation. **b**, Bar chart showing the relative mRNA level of *FWA* in *fwa* and three representative T2 ZF fusion lines using normalized reads of RNA-seq data (RPKM). The error bars indicate the standard error (SE) of the three replicates of each sample. **c**, Flowering time of *fwa*, Col-0 and four representative T2 ZF fusion lines as measured by the number of leaves, the number of plants with 20 or fewer leaves are indicated. **d**, CG, CHG and CHH DNA methylation levels over *FWA* promoter regions in *fwa*, Col-0 and representative T2 ZF fusion lines measured by BS-PCR-seq. Pink vertical boxes indicate ZF binding sites. **e**, The observed/expected values of upregulated (pink bars) and downregulated (dark-blue bars) DEGs over ZF off-target sites in ZF fusion lines, measured by RAD analysis. The asterisks indicate the *P* value calculated with one-sided hypergeometric test, *P* < 0.05: *; *P* < 0.01: **; *P* < 0.001: ***; *P* < 0.0001: ****. The *P* values of each ZF fusion at 100 bp upstream of TSS with downregulated DEGs are 0.07 (EYFP-ZF), 0.02 (ZF-SUVH2), 2.91 × 10^−5^ (MSI1-ZF), 0.004 (LHP1-ZF), 4.43 × 10^−8^ (JMJ14-ZF), 2.09 × 10^−5^ (JMJ18-ZF), 0.001 (HD2A-ZF), 2.79 × 10^−7^ (HD2B-ZF), 9.52 × 10^−9^ (HD2C-ZF), 0.0002 (HDA6-ZF), 4.28 × 10^−7^ (ELF7-ZF) and 0.0006 (CPL2-ZF).

This screen identified 14 effector proteins that successfully restored the early flowering phenotype of the *fwa* epiallele (Fig. 1a). Among these effectors, DMS3, SUVH2, SUVH9 and MORC1 are known players in the RdDM pathway, and previous studies have shown that DMS3-ZF, SUVH9-ZF and MORC1-ZF could restore methylation and silencing of *FWA*^11,12^. SUVH2 is a close homologue of SUVH9 that functions in RdDM^11^, and it was thus not unexpected that ZF-SUVH2 would also methylate and silence *FWA* expression (Fig. 1b–d).

**Extended Data Fig. 1:**
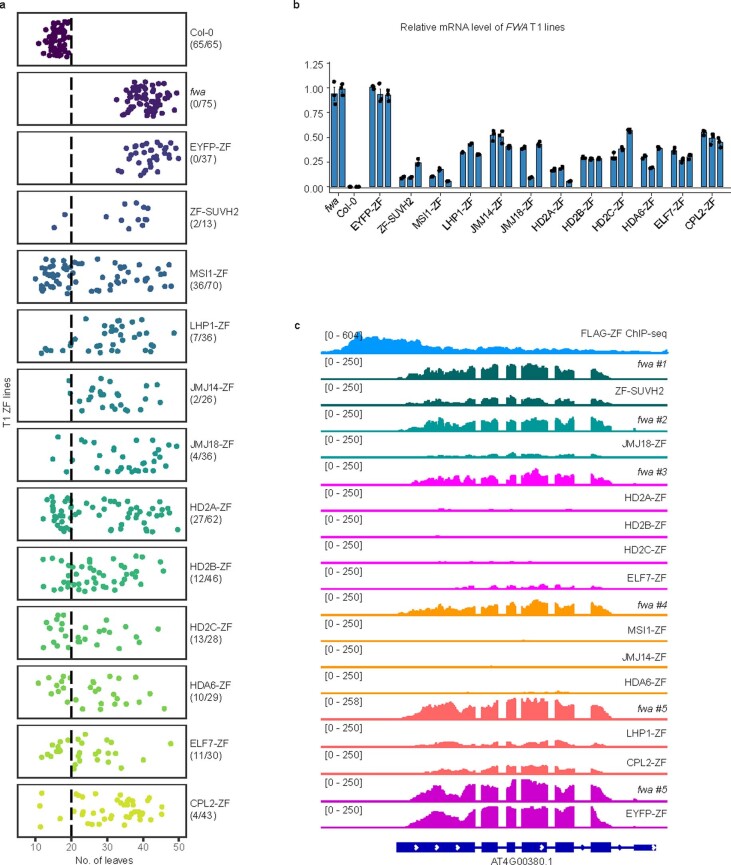
ZF screening identified silencers. **a**, Flowering time of *fwa*, Col-0 and T1 lines of ZF fusions. **b**, qRT–PCR showing the relative mRNA level of *FWA* gene in *fwa*, Col-0 and three representative T1 lines of ZF fusions. Error bars indicate standard error of three technical replicates, data are presented as mean values +/− s.e.m. **c**, Screenshots of RNA-seq signals in *fwa* and representative T2 transgenic lines of ZF fusions over a representative ZF off-target site. The FLAG-ZF ChIP–seq indicates ZF binding site. Source data

We also identified many gene regulators that silenced *FWA* in a DNA methylation-independent manner (Fig. 1a–d), including two polycomb-group proteins MULTICOPY SUPRESSOR OF IRA1 (MSI1) and LIKE HETEROCHROMATIN PROTEIN 1 (LHP1), two JMJs JMJ14 and JMJ18, four HDACs HD2A, HD2B, HD2C and HDA6, a Pol II-associated factor 1 (PAF1) homologue EARLY FLOWERING 7 (ELF7) and Carboxyl-terminal Domain Phosphatase-like 2 (CPL2) (Fig. 1a). The ZF fusions of these proteins restored an early flowering phenotype of the *fwa* epiallele to a similar level as in wild-type Col-0 plants, (Fig. 1c and Extended Data Fig. 1a), even though the *FWA* silencing was less efficient than ZF-SUVH2 (Fig. 1b and Extended Data Fig. 1b).

Although ZF was designed to bind the *FWA* promoter, it also binds to thousands of off-target sites throughout the genome^12^. We therefore performed RNA sequencing (RNA-seq) to determine whether the different ZF fusion proteins could also regulate other genes near these off-target binding sites. We analysed genes near 6,091 ZF chromatin immunoprecipitation followed by sequencing (ChIP–seq) peaks that showed at least four-fold enrichment of ZF ChIP–seq signal relative to a *fwa* non transgenic control (Supplementary Table 2). The differentially expressed genes (DEGs) near ZF off-target peaks were analysed using region-associated DEG (RAD) analysis^20^. This analysis showed that all the ZF fusions, except the control fusion EYFP-ZF, showed a higher number of downregulated DEGs than upregulated DEGs when the ZF peak was within 1 kb of the start site of the gene (Fig. 1e and Extended Data Fig. 1c), suggesting that all the identified fusions can repress many other genes in addition to *FWA*.

Previous studies showed that mutation of some of the effector genes displayed early flowering phenotypes, including *ELF7* (ref. ^21^), *LHP1* (ref. ^22^), *JMJ14* (ref. ^7^) and *CPL2* (ref. ^23^). Therefore, we wanted to ensure that expression of the ZF fusions was not causing a suppression of the expression of the endogenous genes. We compared the expression level of the endogenous gene by examining reads corresponding to the 3′ untranslated region (UTR) regions of the genes, which were excluded from ZF fusion transgenes. We found that the 3′ UTR expression levels were unaltered in the transgenic plants, suggesting that the early flowering phenotype of these ZF fusion lines was due to the silencing of *FWA*, rather than silencing of the endogenous effector encoding genes (Supplementary Fig. 1).

### The heritability of target gene silencing in ZF fusions

**Fig. 2:**
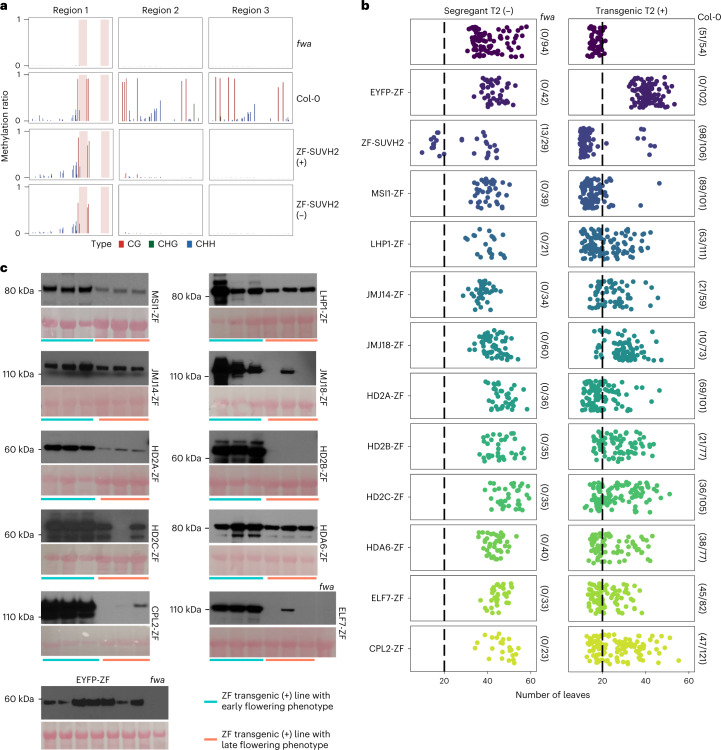
The inheritability of target gene silencing in the T2 lines of ZF fusions. **a**, CG, CHG and CHH DNA methylation levels over *FWA* promoter regions in *fwa*, Col-0, ZF-SUVH2 transgenic T2 line (+) and ZF-SUVH2 segregant T2 line (−), measured by BS-PCR-seq. Pink vertical boxes indicate ZF binding sites. **b**, Flowering time of ZF fusions T2 segregant lines (−) and transgenic lines (+). The number of plants with 20 or fewer leaves are indicated. **c**, Western blot showing the protein expression levels of ZF fusion T2 transgenic lines with early flowering phenotype (left three samples) and late flowering phenotype (right three samples). The top panels of each ZF fusion were blotted with anti-FLAG, and the bottom panels were stained by Ponceau using as loading controls; each experiment was repeated twice independently with similar results. Source data

To test inheritance of the DNA methylation in ZF-SUVH2, bisulfite amplicon sequencing analysis (BS-PCR-seq) was performed to evaluate DNA methylation at *FWA* promoter regions in T2 lines that still contained the transgene and in lines that had segregated away the transgene (null segregants). DNA methylation was observed in both transgenic and null segregant lines, showing that DNA methylation established by ZF-SUVH2 could be heritable in the absence of the transgene (Fig. 2a), as has been shown for other fusion proteins that target DNA methylation to *FWA*^11,12,24^. As expected, the early flowering phenotype was also inherited in many null segregant plants in the T2 population (Fig. 2b and Supplementary Table 3).

We similarly analysed the heritability of the flowering time phenotypes for the ZF fusions that were not associated with *FWA* DNA methylation. We found that, in T2 plants that inherited the fusion protein transgenes, the early flowering phenotype was usually maintained (Fig. 2b). However, in all null segregant plants, the flowering time reverted to the typical late flowering phenotype of *fwa* plants (Fig. 2b), showing that the persistent presence of the fusion protein transgenes was needed for *FWA* silencing. Within the population of transgene-containing T2 plants, we observed wide variation in flowering time (Fig. 2b). This was probably due to differences in the expression level of the fusion proteins, as we observed by western blotting that plants with high levels of transgene expression tended to have an early flowering phenotype, while plants with low protein expression levels tended to have a late flowering phenotype (Fig. 2c).

### Targeted gene silencing by H3K27me3 deposition

**Fig. 3:**
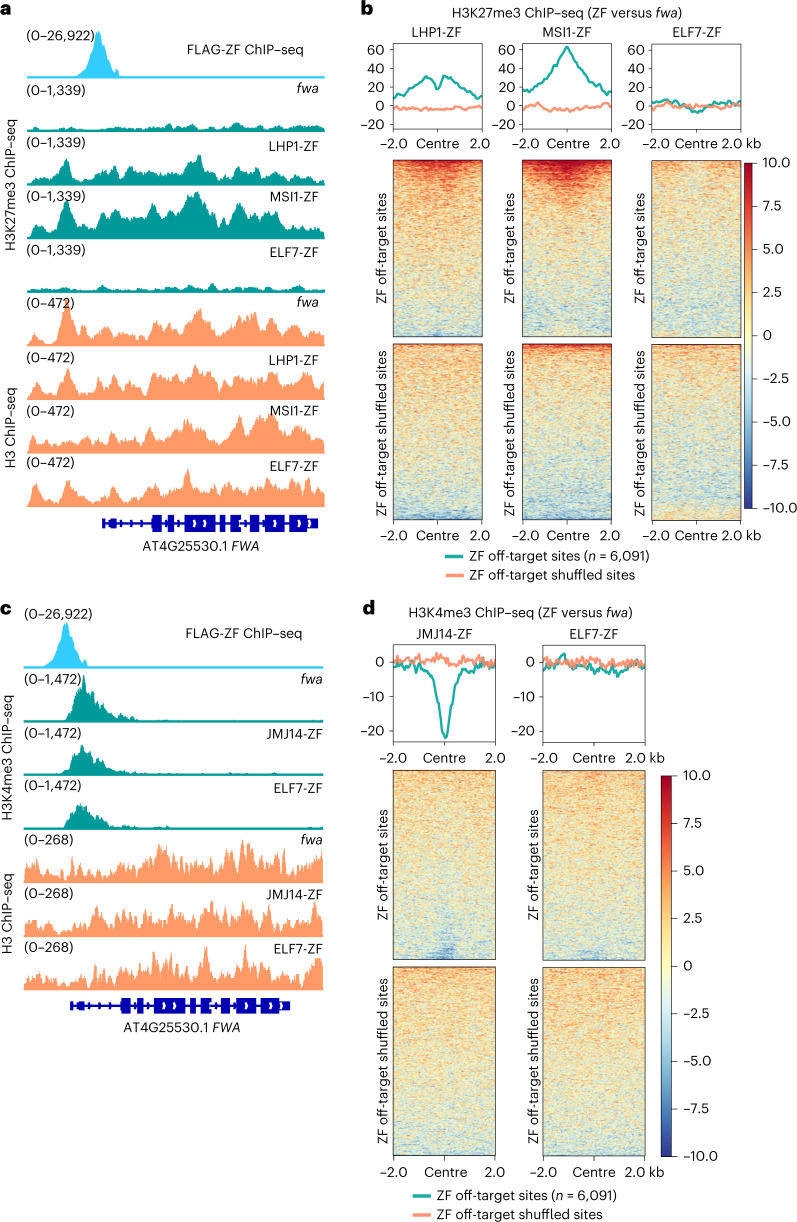
Targeted gene silencing by H3K27me3 deposition and H3K4me3 demethylation. **a**, Screenshots of H3K27me3 (top) and H3 (bottom) ChIP–seq signals over *FWA* region in *fwa*, LHP1-ZF, MSI1-ZF and ELF7-ZF. FLAG-ZF ChIP–seq indicates ZF binding site. **b**, Metaplots and heat maps depicting the normalized H3K27me3 ChIP–seq signals over ZF off-target sites and the shuffled sites (*n* = 6,091) in the representative T2 lines of LHP1-ZF, MSI1-ZF and ELF7-ZF versus *fwa*, respectively. **c**, Screenshots of H3K4me3 (top) and H3 (bottom) ChIP–seq signals over *FWA* region in *fwa*, JMJ14-ZF and ELF7-ZF. The FLAG-ZF ChIP–seq signal indicates ZF binding site. **d**, Metaplots and heat maps depicting the normalized H3K4me3 ChIP–seq signals in the representative T2 lines of JMJ14-ZF and ELF7-ZF versus *fwa* over ZF off-target sites and the shuffled sites (*n* = 6,091), respectively.

**Extended Data Fig. 2:**
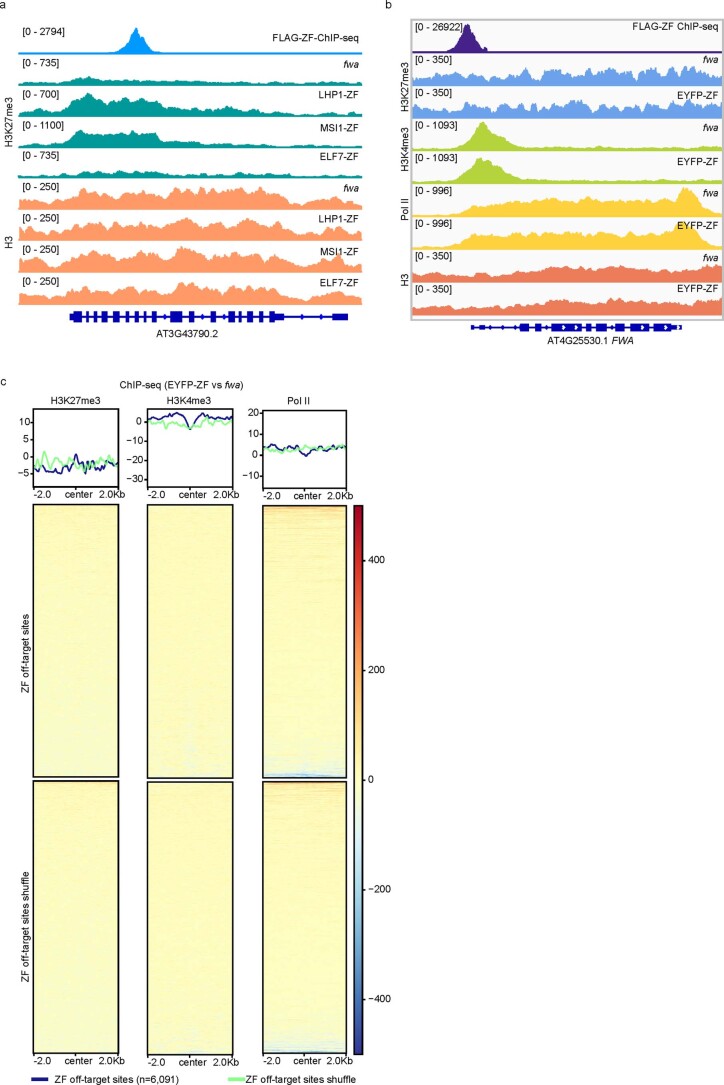
Target gene silencing by histone H3K27me3 deposition. **a**, Screenshots of H3K27me3 and H3 ChIP–seq signals over a representative ZF off-target site in *fwa* and T2 transgenic lines of LHP1-ZF, MSI1-ZF and ELF7-ZF. **b**, Screenshots of H3K27me3, H3K4me3, Pol II Ser5 and H3 ChIP–seq signals in *fwa* and T2 transgenic lines of EYFP-ZF at the *FWA* region. The FLAG-ZF ChIP–seq signals indicate ZF binding sites. **c**, Heat maps and metaplots showing the normalized H3K27me3, H3K4me3, Pol II Ser5 ChIP–seq signals over ZF off-target sites and shuffled sites (*n* = 6,091) in the EYFP-ZF T2 transgenic line versus *fwa*.

MSI1 is a component of the PRC2 complex that also interacts with the *Arabidopsis* PRC2 accessory protein LHP1 (refs. ^25–27^), both of which are important for H3K27me3-mediated gene silencing. Therefore, to test whether H3K27me3 was deposited at *FWA* and ZF off-target sites, H3K27me3 and H3 ChIP–seq were performed in MSI1-ZF, LHP1-ZF and the *fwa* control. Indeed, H3K27me3 ChIP–seq signals were higher at *FWA* in LHP1-ZF and MSI1-ZF than *fwa* control plants (Fig. 3a). We also observed H3K27me3 enrichment in LHP1-ZF and MSI1-ZF when plotting over 6,091 ZF off-target sites (Fig. 3b and Extended Data Fig. 2a), while the H3K27me3 level was similar between a control EYFP-ZF line and *fwa* plants at both *FWA* and ZF off-target loci (Extended Data Fig. 2b,c), suggesting that tethering MSI1 and LHP1 to *FWA* and other target genes can cause gene silencing associated with H3K27me3 deposition.

### Targeted gene silencing by H3K4me3 removal

**Extended Data Fig. 3:**
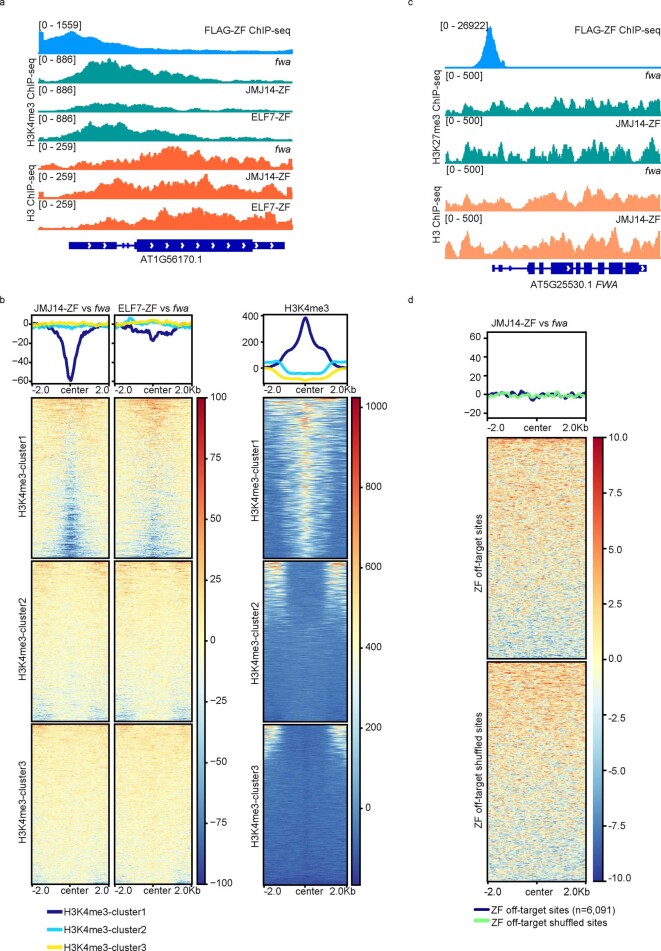
Target gene silencing by H3K4me3 demethylation. **a**, Screenshots of H3K4me3 and H3 ChIP–seq signals over a representative ZF off-target site in *fwa* and T2 transgenic lines of JMJ14-ZF and ELF7-ZF. The FLAG-ZF ChIP–seq signals indicate ZF binding sites. **b**, Heat maps and metaplots showing the normalized H3K4me3 ChIP–seq signals over three clusters of ZF off-target sites in JMJ14-ZF and ELF7-ZF transgenic lines versus *fwa* (left panel). H3K4me3-cluster 1, 2, 3 represent high, medium, and low levels of preexisting H3K4me3 ChIP–seq signals, respectively (right panel). **c**, Screenshots of H3K27me3 and H3 ChIP–seq signals in *fwa* and a representative T2 line of JMJ14-ZF over *FWA*. **d**, Heat maps and metaplot showing the normalized H3K27me3 ChIP–seq signals over ZF off-target sites and shuffled sites (*n* = 6,091) in JMJ14-ZF T2 transgenic line versus *fwa*.

Two H3K4 demethylase protein fusions, JMJ14-ZF and JMJ18-ZF, successfully triggered an early flowering phenotype and silenced *FWA* in a DNA methylation independent manner (Fig. 1a–e). H3K4me3 and H3 ChIP–seq was performed in JMJ14-ZF, where we found that JMJ14-ZF caused a reduction of H3K4me3 over the *FWA* locus (Fig. 3c), as well as over ZF off-target regions (Fig. 3d and Extended Data Fig. 3a), but this was not observed in the EYFP-ZF line (Extended Data Fig. 2b, c). Furthermore, we observed more H3K4me3 removal at ZF off-targets that contained high levels of pre-existing H3K4me3, and less removal at sites with low levels of pre-existing H3K4me3 (Extended Data Fig. 3b). In addition, unlike MSI1-ZF and LHP1-ZF (Fig. 3a,b), JMJ14-ZF did not show accumulation of H3K27me3 at *FWA*, nor at ZF off-target regions (Extended Data Fig. 3c,d). Thus, silencing by JMJ14 is probably acting directly via removal of H3K4me3 rather than by accumulation of H3K27me3, a mark which can act antagonistically with H3K4me3 (refs. ^28–30^). Interestingly, several other H3K4 demethylase proteins were included in our collection, including JMJ16/17, LDL1/2/3 and FLD, but none of these was able to trigger silencing of *FWA*.

### Targeted gene silencing by histone deacetylation

**Fig. 4:**
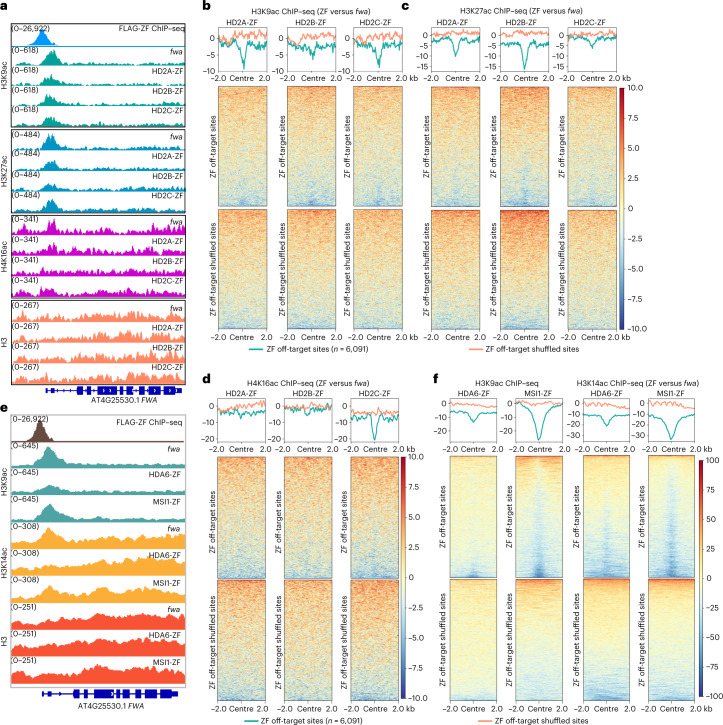
Targeted gene silencing by HDACs and histone deacetylation. **a**, Screenshots of histone H3K9ac, H3K27ac, H4K16ac and H3 ChIP–seq signals over *FWA* region in *fwa*, HD2A-ZF, HD2B-ZF and HD2C-ZF. **b**–**d**, Heat maps and metaplots representing the normalized H3K9ac (**b**), H3K27ac (**c**) and H4K16ac (**d**) ChIP–seq signals over ZF off-target sites and the shuffled sites (*n* = 6,091) in the representative T2 lines of HD2A-ZF, HD2B-ZF and HD2C-ZF versus *fwa*. **e**, Screenshots of histone H3K9ac, H3K14ac and H3 ChIP–seq signals over *FWA* region in *fwa*, HDA6-ZF and MSI1-ZF. **f**, Heat maps and metaplots representing the normalized H3K9ac and H3K14ac ChIP–seq signals over ZF off-target sites and the shuffled sites (*n* = 6,091) in HDA6-ZF and MSI1-ZF T2 representative lines versus *fwa*.

**Extended Data Fig. 4:**
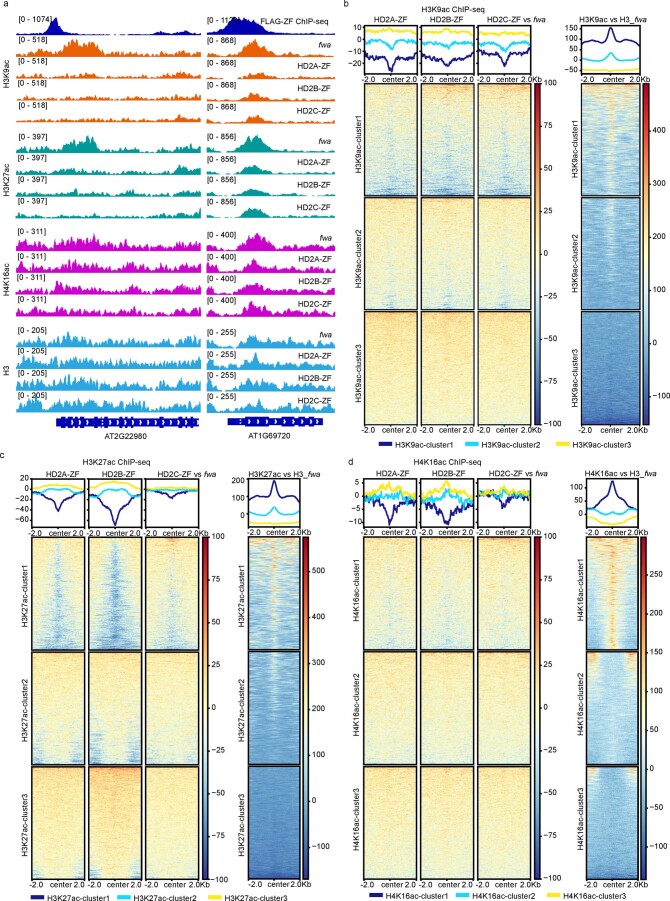
Target gene silencing by HD2-ZFs and histone H3K9, H3K27 and H4K16 deacetylation. **a**, Screenshots of histone H3K9ac, H3K27ac, H4K16ac and H3 ChIP–seq signals over two representative ZF off-target sites in *fwa* and T2 transgenic lines of HD2A-ZF, HD2B-ZF and HD2C-ZF. FLAG-ZF ChIP–seq signals indicate ZF binding sites. **b-d**, Heat maps and metaplots showing normalized H3K9ac (**b**), H3K27ac (**c**) and H4K16ac (**d**) ChIP–seq signals over three clusters of ZF off-target sites in HD2A-ZF, HD2B-ZF and HD2C-ZF versus *fwa* (left panel). H3K9ac (**b**), H3K27ac (**c**), and H4K16ac (**d**) cluster 1, 2, 3 represents high, medium and low levels of preexisting ChIP–seq signals of H3K9ac (**b**), H3K27ac (**c**) and H4K16ac (**d**), respectively (right panel).

Four HDAC proteins, HD2A, HD2B, HD2C and HDA6, were identified from the silencing screen. It was previously shown that *Arabidopsis* HD2A is required for H3K9 deacetylation and ribosomal RNA gene silencing^31^, and that HD2C mediates H4K16 deacetylation and is involved in ribosome biogenesis^32^, suggesting that HD2 family members can deacetylate multiple sites. We performed immunoprecipitation–mass spectrometry (IP–MS) utilizing a pHD2A:HD2A-FLAG transgene, which indicated that all three HD2 type HDACs interact with each other (Supplementary Table 4), consistent with an earlier report of IP–MS of tagged HD2C^32^. In addition, ChIP–seq analysis of pHD2A:HD2A-FLAG plants showed a partial overlap with histone H3K9ac, H3K27ac and H4K16ac in the genome (Supplementary Fig. 2a,b). Thus, we profiled H3K9ac, H4K16ac, H3K27ac and H3 patterns by ChIP–seq in HD2A-ZF, HD2B-ZF, HD2C-ZF (HD2-ZFs) plants. We found that H3K9ac, H4K16ac and H3K27ac were moderately reduced in HD2-ZF plants both at *FWA* (Fig. 4a) and over ZF off-target sites (Fig. 4b–d and Extended Data Fig. 4a), particularly over the loci with a higher level of pre-existing acetylation modifications (Extended Data Fig. 4b–d). However, the acetylation levels were not reduced in EYFP-ZF at both *FWA* and ZF off-target loci (Supplementary Fig. 3a,b).

**Extended Data Fig. 5:**
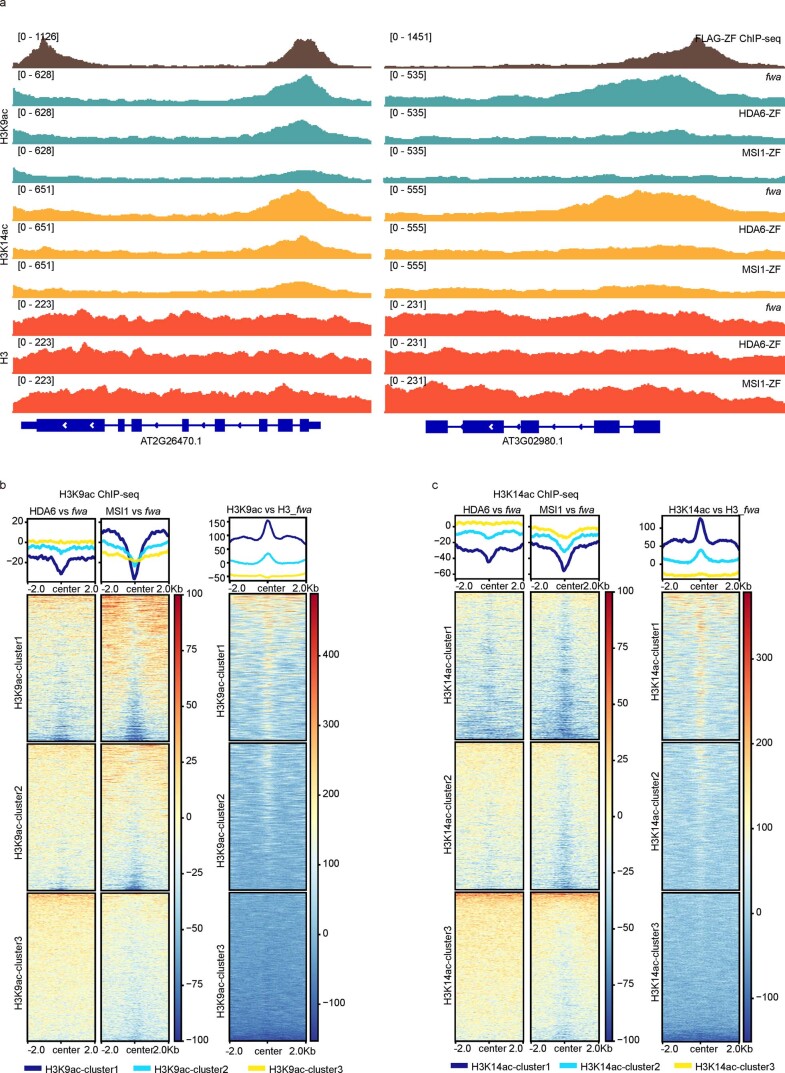
Target gene silencing by HDA6-ZF, MSI1-ZF, and histone deacetylation. **a**, Screenshots indicating H3K9ac, H3K14ac and H3 ChIP–seq signals over two representative ZF off-target sites in *fwa* and T2 transgenic lines of HDA6-ZF and MSI1-ZF. **b-c**, Heat maps and metaplots showing normalized H3K9ac (**b**) and H3K14ac (**c**) ChIP–seq signals over three clusters of ZF off-target sites in HDA6-ZF and MSI1-ZF versus *fwa* (left panel). H3K9ac (**b**) and H3K14ac (**c**) cluster 1, 2, 3 represents high, medium, and low levels of preexisting ChIP–seq signals of H3K9ac (**b**) and H3K14ac (**c**), respectively (right panel).

HDA6 has been reported to deacetylate several substrates including K9, K14, K18, K23 and K27 of the H3 histone tail and K5, K8 and K12 of the H4 histone tail^33^, with H3K9ac and H3K14ac confirmed in multiple studies^34,35^. Moreover, it is known that MSI1 interacts with HDA6 and HDA19, and contributes to histone deacetylation^36,37^. We therefore performed H3K9ac, H3K14ac and H3 ChIP–seq in HDA6-ZF and MSI1-ZF. Indeed, both H3K9ac and H3K14ac ChIP–seq signals were reduced at *FWA* as well as at ZF off-target sites in HDA6-ZF and MSI1-ZF plants (Fig. 4e,f and Extended Data Fig. 5a), particularly over the loci with high levels of pre-existing H3K9ac and H3K14ac (Extended Data Fig. 5b,c). This result suggests that HDA6 and MSI1-ZF repress target gene expression at least partially via histone H3K9 and H3K14 deacetylation. Together our results demonstrate that a variety of different HDAC proteins of different classes can be harnessed for targeted gene silencing. However, since histone acetylation universally goes with gene expression, it is not possible to rule out the possibility that the HDAC-ZF fusions are silencing expression by another mechanism, with the loss of acetylation being an indirect effect of gene silencing^38^.

### Silencing by interference with Pol II transcription

**Extended Data Fig. 6:**
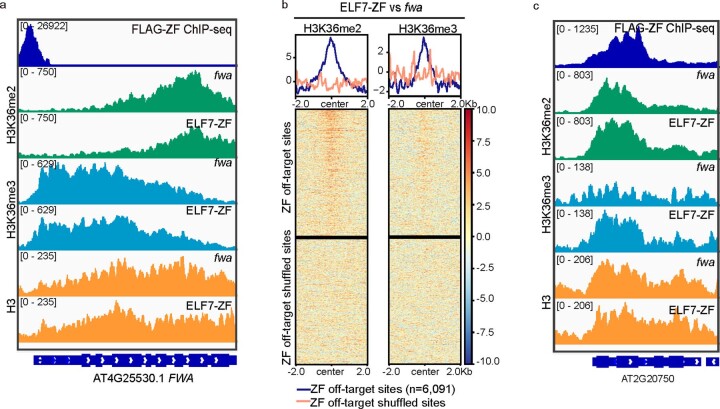
Target gene silencing by ELF7-ZF cannot be explained by H3K36me2/3 alternations. **a**, Screenshots of FLAG-ZF ChIP–seq, and H3K36me2, H3K36me3 and H3 ChIP–seq signals in *fwa* and a representative ELF7-ZF T2 transgenic line over *FWA* region. **b**, Heat maps and metaplots depicting the normalized H3K36me2 (left) and H3K36me3 (right) ChIP–seq signals in ELF7-ZF versus *fwa* over ZF off target sites and shuffled sites (*n* = 6,091). **c**, Screenshots showing the FLAG-ZF ChIP–seq, and H3K36me2, H3K36me3 and H3 ChIP–seq signals in *fwa* and ELF7-ZF over a representative ZF off-target gene.

ELF7 encodes a PAF1 homologue, which is a subunit of the PAF1 complex (PAF1C). PAF1C is a conserved protein complex in eukaryotes that collaborates with Pol II during transcription initiation and elongation^39,40^. In *Arabidopsis*, mutation of PAF1C subunit *VIP3* caused a redistribution of histone H3K4me3 and H3K36me2 in certain genes^41^, and we therefore initially performed H3K4me3, H3K36me2 and H3K36me3 ChIP–seq to determine whether changes in these epigenetic marks might explain ELF7-ZF triggered *FWA* suppression. We observed some reduction of H3K4me3 at *FWA* in ELF7-ZF compared with *fwa* control plants (Fig. 3c). However, unlike JMJ14-ZF, H3K4me3 signal was largely unaffected near ZF off-target sites in ELF7-ZF (Fig. 3d). Considering that ELF7-ZF did trigger gene silencing at ZF off-target sites (Fig. 1e and Extended Data Fig. 1c), it seemed unlikely that H3K4me3 reduction was the relevant mechanism. In addition, signals of both H3K36me2 and H3K36me3 were slightly decreased at the *FWA* locus (Extended Data Fig. 6a), while at the same time somewhat increased over ZF off-target sites (Extended Data Fig. 6b,c), making it unlikely that changes in H3K36me2 or H3K36me3 levels were the direct cause of ELF7-ZF-mediated gene silencing.

**Fig. 5:**
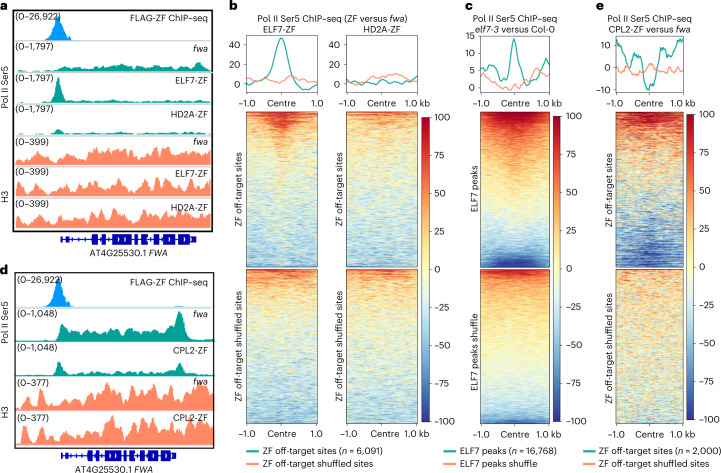
Targeted gene silencing by ELF7, CPL2 and Pol II transcription disruption. **a**, Screenshots of Pol II Ser5 and H3 ChIP–seq signals over *FWA* region in *fwa*, ELF7-ZF and HD2A-ZF. **b**, Heat maps and metaplots representing normalized Pol II Ser5 ChIP–seq signals in the representative T2 lines of ELF7-ZF and HD2A-ZF versus *fwa*, over ZF off-target sites and the shuffled sites (*n* = 6,091), respectively. **c**, Heat map and metaplot showing normalized Pol II Ser5 ChIP–seq signals over ELF7-FLAG ChIP–seq peaks (*n* = 16,768) and ELF7 peaks shuffle in *elf7-3* versus Col-0. **d**, Screenshots of Pol II Ser5 ChIP–seq signals over *FWA* in *fwa* and a CPL2-ZF representative T2 transgenic line. **e**, Heat map and metaplot depicting the normalized Pol II Ser5 ChIP–seq signals over ZF off-target sites that had pre-existing Pol II Ser5 ChIP–seq signals (*n* = 2,000) and the shuffled sites in CPL2-ZF versus *fwa*.

**Extended Data Fig. 7:**
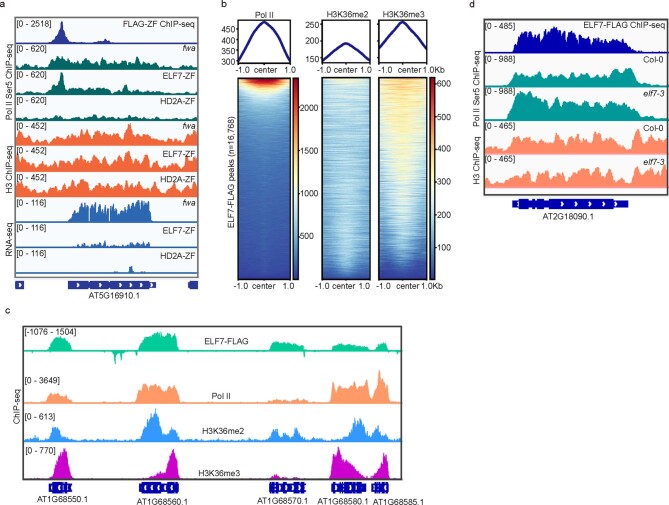
Target gene silencing by ELF7-ZF. **a**, Screenshots of Pol II Ser5 and H3 ChIP–seq and RNA-seq signals over a representative ZF off-target site in *fwa*, ELF7-ZF, and HD2A-ZF. **b**, Heat maps and metaplots showing Pol II Ser5, H3K36me2 and H3K36me3 ChIP–seq signals over ELF7-FLAG ChIP–seq peaks (*n* = 16,768) in Col-0. **c**, Screenshots of ELF7-FLAG, Pol II Ser5, H3K36me2 and H3K36me3 ChIP–seq signals over five representative ELF7 binding genes. **d**, Screenshots of ELF7-FLAG, Pol II Ser5 and H3 ChIP–seq signals in Col-0 and *elf7-3* mutants over a representative ELF7 targeting gene.

We generated pELF7:ELF7-FLAG complementing transgenic lines in the *elf7-3* mutant background to perform IP–MS to identify ELF7-interacting proteins^21^ (Supplementary Fig. 4). Consistent with previous work^40^ our ELF7 IP–MS data identified peptides corresponding to all of the subunits of the PAF1C, as well as Pol II subunits and transcription factors, consistent with a role of ELF7 in Pol II transcription (Supplementary Table 4). Since ELF7 is a Pol II-interacting protein, we hypothesized that ELF7-ZF might interact with Pol II at the *FWA* promoter region, retaining it there and inhibiting transcription. To test this, we performed Pol II serine 5 (Ser5) ChIP–seq in ELF7-ZF transgenic lines, as well as *fwa*, EYFP-ZF and HD2A-ZF as controls. As expected, Pol II occupancy at *FWA* transcribed regions was strongly reduced in ELF7-ZF, as well as in HD2A-ZF (Fig. 5a), but not in EYFP-ZF (Extended Data Fig. 2b,c), consistent with the silencing of *FWA* expression in these lines (Fig. 1b). However, we observed a very prominent Pol II peak at the *FWA* promoter overlapping the ZF site in the ELF7-ZF line, but not in HD2A-ZF, EYFP-ZF or *fwa* plants (Fig. 5a and Extended Data Fig. 2b,c). Moreover, strong Pol II enrichment was also observed at the ZF off-target binding sites but not the shuffled controls sites in ELF7-ZF (Fig. 5b and Extended Data Fig. 7a). Thus, Pol II appears to be tethered to the ZF binding sites due to the interaction with ELF7-ZF, which in turn appears to inhibit Pol II transcription, leading to gene silencing.

To better understand the endogenous function of ELF7, we performed ChIP–seq in pELF7:ELF7-FLAG transgenic lines. Consistent with its role in transcriptional elongation, ELF7 was exclusively distributed over gene body regions, with most ELF7 signals overlapping with both Pol II peaks and H3K36me2 or H3K36me3 peaks (Extended Data Fig. 7b,c). We also performed Pol II Ser5 ChIP–seq in *elf7-3* and found a notable accumulation of Pol II at ELF7 enriched sites but not the shuffled control sites^42^ (Fig. 5c and Extended Data Fig. 7d), suggesting that transcriptional elongation is impeded, resulting in a higher Pol II occupancy reflected in the ChIP–seq data. Together our data show that the *Arabidopsis* PAF1C is required for proper Pol II transcriptional elongation, as has been shown in yeast and animal systems^43,44^, and that tethering the ELF7 component of the complex to promoters represents a novel synthetic mechanism to induce gene silencing that is probably independent of changes of particular epigenetic marks.

### Target gene silencing by Pol II CTD Ser5 dephosphorylation

CPL2 is a well-characterized phosphatase that specifically acts on Ser5 of the Pol II C-terminal domain^45^, and represses transcription through inhibiting Pol II activity^46,47^. We therefore performed Pol II Ser5 ChIP–seq in CPL2-ZF transgenic lines, and we indeed observed reduced signal at *FWA* as well as ZF off-target sites that had pre-existing Pol II Ser5 (Fig. 5d,e and Supplementary Fig. 5), suggesting that CPL2-ZF indeed silenced target genes through Pol II CTD Ser5 dephosphorylation, although our data do not allow us to pinpoint the exact mechanism of repression. The promoter tethering of CPL2 thus represents a new mechanism for targeted gene silencing.

### Target genes vary widely in different ZF fusions

**Fig. 6:**
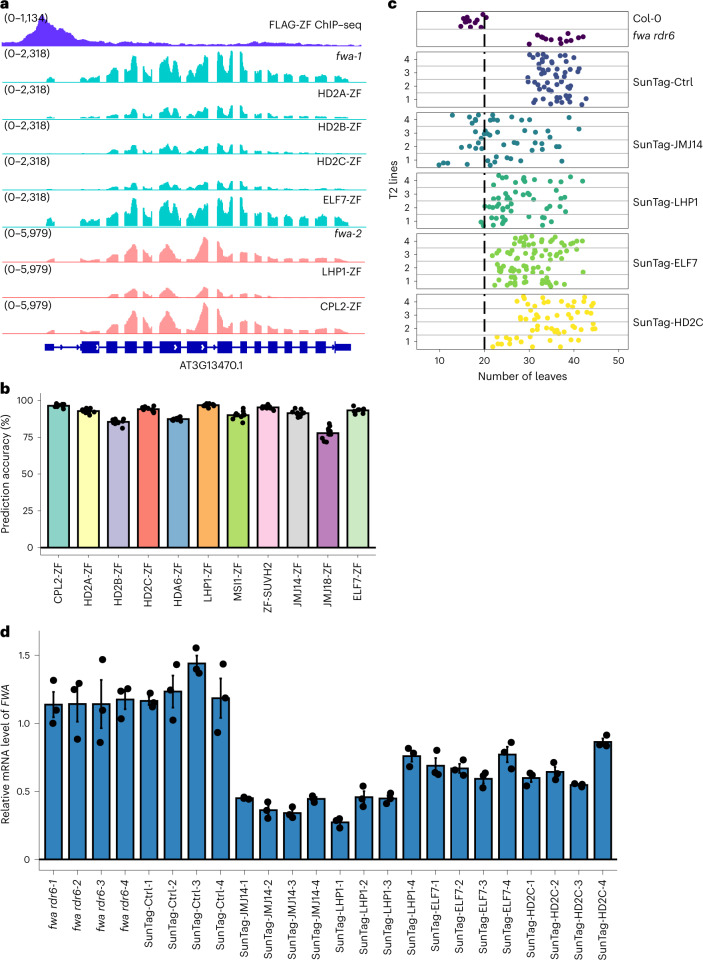
Predicting the efficacy of different effector proteins by using machine learning models and some effector protein also worked in SunTag system. **a**, Screenshots displaying RNA-seq levels over a representative ZF off-target gene AT3G13470 in *fwa-1*, HD2A-ZF, HD2B-ZF, HD2C-ZF, ELF7-ZF, *fwa-2*, LHP1-ZF and CPL2-ZF. FLAG-ZF ChIP–seq indicates the binding site of ZF. **b**, Bar chart indicating the accuracy of ten-fold cross-validation in each ZF line. **c**, Dot plots displaying leaf numbers of Col-0, *fwa rdr6* and representative SunTag T2 lines. **d**, qRT–PCR analysis showing the relative mRNA level of *FWA* in Col-0, *fwa rdr6* and representative SunTag T2 lines. Error bars indicate the standard error of the mean (s.e.m.) of three technical replicates of each sample; data are presented as mean ± s.e.m.

**Extended Data Fig. 8:**
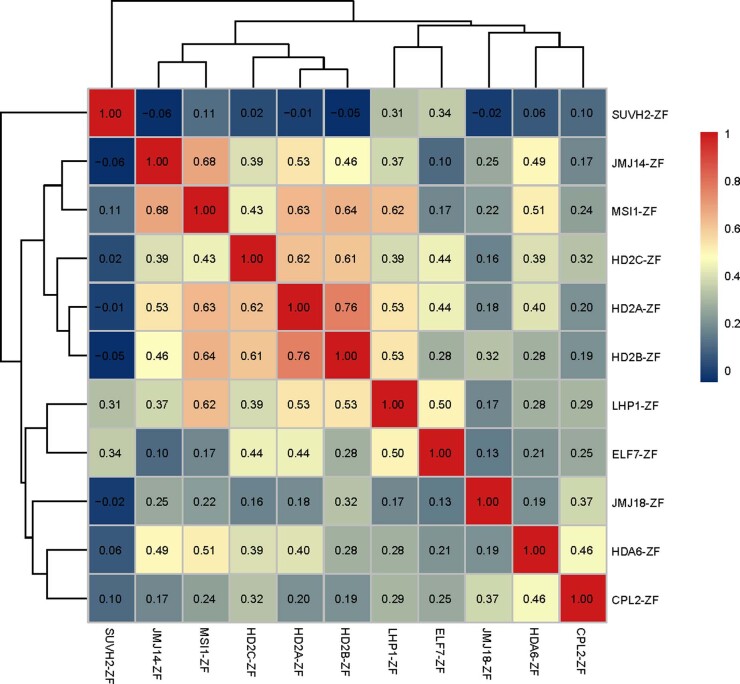
Overall overlapping of the target genes among all the ZF fusions. Heat map displaying Spearman correlation coefficients for all the down regulated ZF target genes (distance to TSS is from −500 to 200 bp, *n* = 805) among all the ZF fusions.

**Extended Data Fig. 9:**
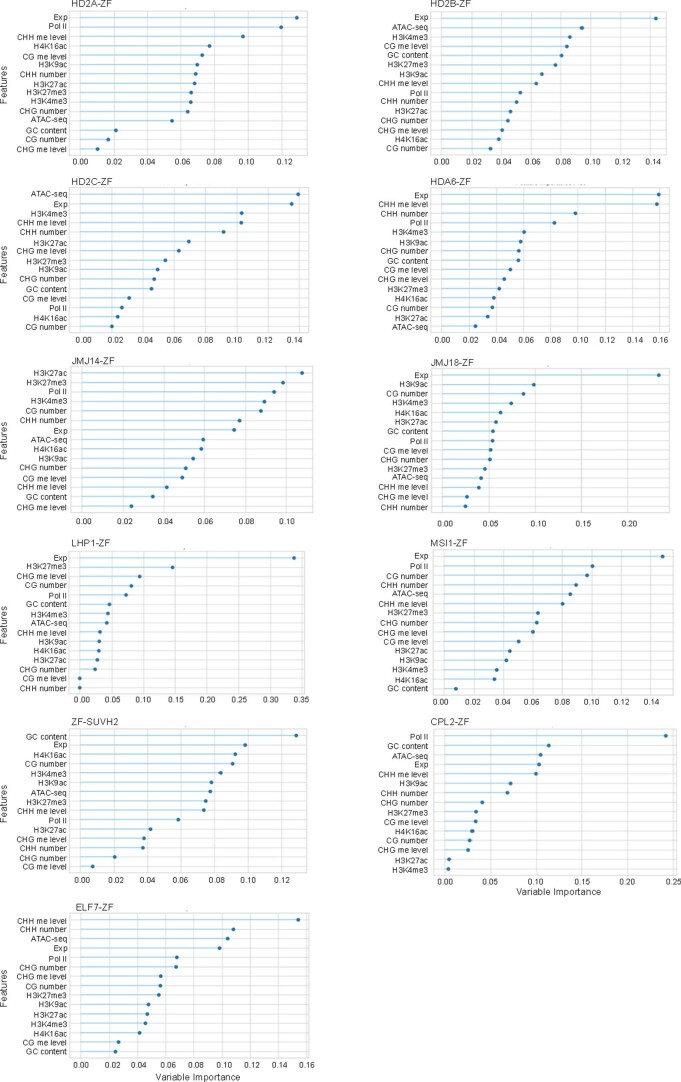
The importance of varied chromatin features contributed to the modeling construction by machine learning. The line point charts displaying the variable importance of different chromatin features of ZF lines as inputs for machine learning.

We observed that the set of downregulated genes at ZF off-target sites for each ZF fusion were partially non-overlapping (Extended Data Fig. 8). As one example, gene AT3G13470 was downregulated by HD2A-ZF, HD2B-ZF, HD2C-ZF and LHP1-ZF, but not ELF7-ZF nor CPL2-ZF (Fig. 6a). These results suggest that the best gene silencing approach will greatly depend on the particular target gene of interest, highlighting the utility of gene silencing tools that work by different mechanisms. We hypothesized that pre-existing epigenetic features of the target genes might determine their sensitivity to silencing by the different ZF fusion proteins, and indeed different target genes showed different levels of various epigenetic marks including histone acetylation (H3K9ac, H3K27ac and H4K16ac), histone methylation (H3K4me3 and H3K27me3), chromatin accessibility (assay for transposase-accessible chromatin using sequencing (ATAC-seq)) and DNA methylation (Supplementary Fig. 6 and Supplementary Table 5). To test this hypothesis, we used the chromatin features of 2,709 genes containing nearby ZF binding sites, as well as the expression level of these genes, together with the information of whether these genes were silenced with each of the ZF fusion as inputs to various machine learning algorithms (Supplementary Table 5). The decision tree classifier showed an excellent performance, and a ten-fold cross-validation of this model showed an extremely high accuracy (78–96%) in predicting the efficacy of each silencer (Fig. 6b and Supplementary Table 6). In addition, the importance of each feature to the model construction was also revealed by this method (Extended Data Fig. 9). Notably, the target gene expression level was common feature that contributed to almost every silencer, Pol II ChIP–seq played a dominant role in CPL2-ZF, and the rest of the silencers were usually determined by a combination of varied features (Extended Data Fig. 9), supporting the hypothesis that different effector proteins are efficient in silencing genes with different chromatin features.

### Some effector proteins were effective in a dCas9-SunTag system

**Extended Data Fig. 10:**
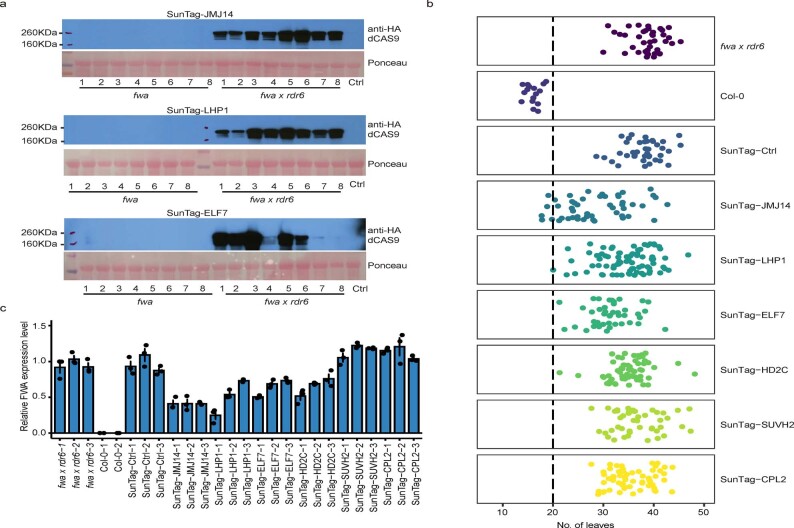
Some effector also triggered gene silencing in SunTag system. **a**, Western blots showing the expression level of dCAS9 in SunTag-JMJ14, LHP1 and ELF7 under *fwa* and *fwa rdr6* background, respectively. **b**, Flowering time of *fwa rdr6*, Col-0 and SunTag T1 transgenic lines, measured by leaf number. **c**, RT-qPCR results showing the relative mRNA level of *FWA* in *fwa rdr6*, Col-0 and SunTag T1 transgenic lines. Error bars indicate the mean SE of three technical replicates of each sample.

We cloned SUVH2, JMJ14, LHP1, HD2C, ELF7 and CPL2, representing different epigenetic pathways targeting gene silencing, into the SunTag system. The SunTag is a dCas9-based system in which dCas9 is fused to a chain of peptide epitopes, with effector proteins fused in a separate cassette with a single-chain antibody recognizing the peptide epitopes, such that a single dCas9 can recruit multiple effector proteins^14,16^. All the effectors in the SunTag system initially failed to trigger gene silencing or an early flowering phenotype in the *fwa* background, which we hypothesized was due to gene silencing of the transgene since western blot analysis showed little expression of dCas9 (Extended Data Fig. 10a). To reduce silencing, we retransformed all constructs into the *fwa rdr6* genetic background^48^, and found that this dramatically increased the expression of dCas9 (Extended Data Fig. 10a). In this background we observed target *FWA* gene silencing and an early flowering phenotype in the JMJ14 and LHP1 lines, and to a lesser extent in ELF7 and HD2C, but not in SUVH2, CPL2 or the no effector SunTag control (Fig. 6c,d and Extended Data Fig. 10b,c). These results indicate that that some effector proteins identified from this work can be also used for target gene silencing via the SunTag system.

## Discussion

By constructing ZF fusions to target a collection of putative chromatin regulators to *FWA*, we uncovered a variety of proteins capable of inducing gene silencing at *FWA* as well as at many other loci. This screen was done in the *fwa* epiallele background in which there are no short interfering RNAs, H3K9me2, RdDM components or DNA methylation at the target locus. Further, most of the silencers described here functioned without inducing any of these heterochromatin-related epigenetic marks. Our genomic and genetic evidence demonstrate that target gene silencing can be directed by diverse histone modifications including H3K27me3 deposition, H3K4me3 demethylation and histone deacetylation at H3K9, H3K14 and H4K16 tails. Interestingly, we also found two factors that appear to act by more directly interacting with Pol II, the ELF7 component of the elongation factor PAF1C and the CPL2 enzyme, which dephosphorylates the RNA Pol II C-terminus.

Due to protein–protein interactions between these effectors with the other epigenetic regulators, it is possible that some effectors might silence target genes through combined mechanisms. For example, it is known that HD2A, B and C interacts with HDA6^49^, which might deacetylate different histone tail residues. As another example, MSI1 interacts with HDA19, HISTONE DEACETYLATION COMPLEX1 (HDC1), HDA6, SIN3-like proteins and other proteins^36,37^, and our results indicated that MSI1-ZF might trigger gene silencing via a combination of H3K27me3 deposition and histone deacetylation (Fig. 4e,f and Extended Data Fig. 5a). This could also explain why MSI1-ZF silenced more efficiently than LHP1-ZF, HDA6-ZF and HD2-ZFs at *FWA* (Fig. 1b). The differences in interacting proteins between MSI1 and LHP1 might also explain why they silenced different sets of target genes (Supplementary Table 7).

Our results show that diverse pathways can be harnessed for the development of synthetic biology tools to downregulate genes. DNA methylation represents a strong and potentially heritable type of silencing, but only some genes will be amenable to this type of modification due to low densities of CG dinucleotides that are needed for silencing and heritability^12^ or high levels of endogenous expression that can compete with DNA methylation maintenance^14^. In addition, it may often be desirable to cause only a partial silencing of a target gene, and the non-DNA-methylation-based tools described here were able to cause various levels of repression that were lower than that of DNA methylation targeting tools (Fig.1b).

Our data showed that certain genes were more amenable to particular gene silencing approaches, which is probably due to differences in proximal chromatin environments for different target genes. Therefore, having a wider array of silencing tools expands the range of genes that can be successfully targeted. This also suggests that a different set of proteins from the library might have been identified if we had utilized a different reporter gene other than *FWA*, or if we had targeted a position other than the promoter region of the reporter gene. The genes susceptible to silencing in this study were involved in a wide range of biological processes including development, hormone signalling pathways and disease resistance (Supplementary Table 7), suggesting that the tools described here could be useful for the modulation of many different traits. In addition, we were able to utilize machine learning algorithms that could accurately predict which genes would be sensitive to silencing by a particular gene silencing tool. The prediction was extended to all *Arabidopsis* genes (Supplementary Table 6), which may be useful for future gene expression engineering efforts. In conclusion, this work provides mechanistic detail for an array of key plant gene silencing pathways, and describes a collection of new tools that should be useful in both basic research and crop improvement.

## Methods

### Plant materials and growth conditions

All the plants used in this paper were in the *Arabidopsis thaliana* Col-0 ecotype, grown under long-day conditions (16 h light and 8 h dark). The T-DNA insertion lines used in this study included *elf7-3* (SALK_019433)^42^. All the transgenic plants were generated by *Agrobacterium* (AGL0 strain)-mediated floral dipping.

### Plasmid construction

pMDC123-UBQ10: ZF-3xFLAG-effector (cDNA) is a Gateway compatible binary destination vector that includes a plant UBQ10 promoter, followed by an N-terminal ZF and 3xFLAG epitope tag, a Gateway cassette and an OCS terminator. The list of selected effectors from *Arabidopsis* Gene ORFeome Collection (Supplementary Table 1) in the pENTR/D-TOPO vectors were all cloned into pMDC123 destination vector via LR reaction using Gateway LR Clonase II (Invitrogen). pEG302-effector (gDNA)-3xFLAG-ZF is also a Gateway-compatible binary destination vector, which consists of a gateway cassette, followed by a C-terminal 3xFLAG epitope tag, a ZF, a Biotin Ligase Recognition Peptide and an OCS terminator. The sequence of native promoter (~1.5 kb upstream from the 5′ UTR or until the next gene annotation) and genomic DNA (without stop codon) of the effectors were cloned into pENTR D-TOPO vectors (Invitrogen), which were used to deliver the genomic DNA sequences of these effectors into the destination vector using Gateway LR Clonase II (Invitrogen). pEG302-effector (gDNA)-3xFLAG/9xMyc contains a gateway cassette, followed by a C-terminal 3xFLAG or 9xMyc epitope tag, a Biotin Ligase Recognition Peptide and an OCS terminator. The cloning method is the same as pEG302-effector (gDNA)-3xFLAG-ZF. For the SunTag-effector, the catalytic domain of JMJ14 and the coding sequence of other effector proteins were cloned into the SunTag vector with the infusion method (Takara). Please see Supplementary Table 8 for the primers and guide RNA information for the SunTag system.

### Flowering time measurement

The flowering times were measured by the leaf counts, and each dot in the dot plots represents the leaf number of individual plant. The number of plants with 20 or fewer leaves were marked.

### BS-PCR-seq

The leaf tissue from 4- to 5-week-old Col-0 wild type, *fwa* and the representative T2 ZF lines showing early flowering phenotype were collected to perform BS-PCR at *FWA* promoter regions. Cetyltrimethyl ammonium bromide-based method was used to extract DNA, and the EpiTect Bisulfite kit (QIAGEN) was used for DNA conversion. The converted DNA was used as a template to amplify three different regions over promoter and 5′ transcribed regions of *FWA*, including region 1 (chr4: 13038143-13038272), region 2 (chr4: 13038356- 13038499) and region 3 (chr4: 13038568-13038695). Pfu Turbo Cx (Agilent), dNTP (Takara Bio) and the primers designed for the above-mentioned *FWA* regions (Supplementary Table 8) were used to perform PCR reactions. Three different PCR products from three regions of each sample were pooled and purified with AMPure beads (Beckman Coulter). The purified PCR products were used to construct libraries by the Kapa DNA Hyper Kit (Roche) together with TruSeq DNA UD indexes for Illumina (Illumina), and the libraries were sequenced on Illumina iSeq 100.

### IP–MS

The method of IP–MS used in this study has been described in a recent paper^13^. Ten grams of unopened floral buds from Col-0 wild type and FLAG-tag transgenic plants HD2A and ELF7 were collected and ground into fine powder with liquid nitrogen. These samples were resuspended with 25 ml IP buffer and homogenized until lump-free by Dounce homogenizer. The lysate was filtered through Miracloth and incubated with 250 μl anti-FLAG M2 magnetic beads (Sigma) at 4 °C for 2 h. The magnetic beads were washed with IP buffer and eluted with TBS containing 250 µg ml^−1^ 3xFLAG peptides. The eluted proteins were precipitated with trichloroacetic acid (Sigma) and subject to MS analyses as described previously^13^.

### ChIP–seq

We followed a previous protocol for ChIP–seq with minor modifications^11^. Five microlitres of anti-H3K9ac (ab4441, Abcam), anti-H3K14ac (ab52946, Abcam), anti-H3K27ac (ab52946, Abcam), anti-H4K16ac (ab109463, Abcam), anti-Pol II Ser5 (ab5131, Abcam), anti-H3K36me2 (ab9049, Abcam), anti-H3K36me3 (ab9050, Abcam), anti-H3K4me3 (04-745, Millipore) or anti-H3K27me3 (07-449, Millipore) or 10 μl of anti-FLAG (Sigma) have been added into each ChIP, accordingly. Briefly, a total of 2–4 g of the leaves of T2 ZF lines with early a flowering phenotype or unopened flower buds of the FLAG lines were collected. The plant materials were ground with liquid nitrogen and fixed with 1% formaldehyde containing nuclei isolation buffering for 10 min before adding fresh-made glycine to terminate the crosslinking reaction. The nuclei were isolated and disrupted by SDS-containing lysis buffer, and the chromatin was sheared via Bioruptor Plus (Diagenode) and immunoprecipitated with antibody at 4 °C overnight. Next, the magnetic Protein A and Protein G Dynabeads (Invitrogen) were added and incubated at 4 °C for 2 h. After washing and elution, the reverse crosslinking was done at 65 °C overnight. Then the protein–DNA complex was treated with Protease K (Invitrogen) at 45 °C for 4 h, and the DNA was purified and precipitated with 3 M sodium acetate (Invitrogen), GlycoBlue (Invitrogen) and ethanol at −20 °C overnight. The precipitated DNA was directly used for library construction using the Ovation Ultra Low System V2 kit (NuGEN), and the libraries were sequenced on Illumina NovaSeq 6000 or HiSeq 4000 instruments.

### RNA-seq

Leaf tissue of 4- to 5-week-old plants with similar age from *fwa* and early flowering ZF transgenic lines was collected for RNA extraction using Direct-zol RNA MiniPrep kit (Zymo Research). One microgram of total RNA was used to prepare the libraries for RNA-seq following TruSeq Stranded mRNA kit (Illumina), and the libraries were sequenced on Illumina NovaSeq 6000 or HiSeq 4000 instruments.

### BS-PCR-seq analysis

BS-PCR-seq data analysis in this study used the pipeline described in ref. ^12^. The raw pair-end sequencing reads of each sample were combined, and aligned to both strands of reference genome TAIR10 using BSMAP (v.2.90)^50^, and the alignment allowed up to two mismatches and one best hit. The reads with fewer than 20 reads coverage of cytosines and the reads with more than three consecutives methylated CHH sites were removed. The methylation level of each cytosine was calculated using the ratio of mC/(C + T), and only the methylation data within the designed *FWA* regions were kept for making a plot using customized R scripts.

### ChIP–seq analysis

The ChIP–seq raw reads were trimmed using trim_galore (v0.6.5) (https://www.bioinformatics.babraham.ac.uk/projects/trim_galore/) and then aligned to TAIR10 genome using Bowtie2 (v2.1.0) (ref. ^51^), which allowed one unique mapping site and zero mismatch. The Samtools version 1.9 (ref. ^52^) was used to remove the duplicated reads, and together with deeptools version 3.1.3 (ref. ^53^) to generate tracks using reads per kilobase of exon per million reads mapped (RPKM) for the normalization. The peaks were called using MACS2 version 2.1.1 (ref. ^54^), and the peaks that were frequently existed in previous FLAG ChIP–seq of Col-0 were removed.

For FLAG-ZF ChIP–seq, the FLAG ChIP–seq was performed in the unopened flower buds of FLAG-ZF T2 transgenic plants and *fwa* plants. The peaks were called by FLAG-ZF against *fwa*, and the peaks with four-fold or higher signal enrichment were kept as the ZF off-target sites (Supplementary Table 2, *n* = 6,091), while the other FLAG ChIP–seq used signal enrichment of two-fold or higher for the following analysis.

For the comparison of histone and Pol II (histone/Pol II) enrichment over ZF off-target sites between ZF lines and *fwa*, the histone/Pol II ChIP–seq of each sample including both ZF lines and *fwa* was normalized with their respective H3 ChIP–seq first by using bigwigCompare, and then the normalized histone/Pol II ChIP–seq of ZF lines were further normalized to *fwa* by using bigwigCompare, which were then used to make the metaplot over ZF off-target peaks and random shuffled peaks. This method was also applied in Pol II ChIP–seq enrichments between Col-0 wild type and *elf7-3* mutant over ELF7 peaks and shuffled peaks (Fig. 5c).

### RNA-seq analysis

The RNA-seq raw reads were aligned to TAIR10 genome using Bowtie2 (v2.1.0) (ref. ^51^), and the expression levels were calculated with rsem-calculate-expression from RSEM (v1.3.1) with default settings^55^. The RNA-seq tracks were generated using Samtools version 1.9 (ref. ^52^) and normalized with RPKM using bamCoverage from deeptools version 3.1.3 (ref. ^53^). The DEGs were called using customized scripts of run_DE_analysis.pl from Trinity version 2.8.5 (ref. ^56^). Log_2_ fold change ≥1 and false discovery rate <0.05 were used as a cut-off.

The method of RAD analysis in Fig. 1e is described at ref. ^20^. We used the FLAG-ZF ChIP–seq peaks (*n* = 6,091) as input of the favourite regions, and up- and downregulated DEGs of ZF lines versus *fwa* were used, respectively, as the inputs of DEGs.

### Machine learning

#### ZF targeting genes

We defined 6,091 ZF offtarget sites above; however, not all of them were located proximal to genes. Therefore, we considered 2,709 genes as ZF targeting genes, whose transcription start sites (TSSs) were located between −500 bp and 200 bp from a ZF peak. The chromatin features of these 2,709 ZF target sites as well as the gene expression level of ZF target genes were subjected to the following analysis.

#### Input data preparation

A total of 15 chromatin or genomic features of the 2,709 ZF off-target sites/genes, including gene expression level, ChIP–seq signals of H3K27ac, H3K27me3, H3K4me3, H4K16ac, H3K9ac and Pol II, ATAC-seq signals, the level of CG, CHG and CHH methylation, the number of CG, CHG and CHH sites, and GC content, that have been characterized in the *fwa* epiallele (Supplementary Table 5) were utilized to generate input data for machine learning. We also used a binary gene classification based on whether the gene was downregulated (fold change ≤2 and false discovery rate <0.05) in ZF lines compared with the *fwa* epiallele plants. Input data were pre-processed by removing perfect collinearity.

#### Prediction model selection

We evaluated 14 machine learning algorithms with ten-fold cross-validation (Fig. 6b) in PyCaret (v2.3.4, https://pycaret.org/) to select prediction models. The input dataset was divided into ten groups, with nine groups used as training dataset and one group as test data, reiterated ten times. The decision tree classifier method was applied in modelling as this method showed good performance and also provided the contribution of each feature to the model construction.

### Reporting summary

Further information on research design is available in the Nature Portfolio Reporting Summary linked to this article.

## Supplementary information

Supplementary Information Supplementary figs. 1–6.

Reporting Summary

Supplementary Table Supplementary tables 1–8.

## Data Availability

The accession number for all the high-throughput sequencing data of this paper is GEO: GSE197063. Source data are provided with this paper.

## Source data

Source Data Fig. 2 Unprocessed western blots of Fig. 2c.
Source Data Extended Data Fig./Table 1 Unprocessed western blots of Extended Data Fig. 1a.

## References

[1] Feng S, Jacobsen SE, Reik W (2010). Epigenetic reprogramming in plant and animal development. Science.

[2] Law JA, Jacobsen SE (2010). Establishing, maintaining and modifying DNA methylation patterns in plants and animals. Nat. Rev. Genet..

[3] Kinoshita T (2004). One-way control of FWA imprinting in *Arabidopsis* endosperm by DNA methylation. Science.

[4] Mozgova I, Hennig L (2015). The polycomb group protein regulatory network. Annu. Rev. Plant Biol..

[5] Jiang D, Wang Y, Wang Y, He Y (2008). Repression of FLOWERING LOCUS C and FLOWERING LOCUS T by the *Arabidopsis* polycomb repressive complex 2 components. PLoS ONE.

[6] Mozgova I, Kohler C, Hennig L (2015). Keeping the gate closed: functions of the polycomb repressive complex PRC2 in development. Plant J..

[7] Lu F, Cui X, Zhang S, Liu C, Cao X (2010). JMJ14 is an H3K4 demethylase regulating flowering time in *Arabidopsis*. Cell Res..

[8] Liu C, Lu F, Cui X, Cao X (2010). Histone methylation in higher plants. Annu. Rev. Plant Biol..

[9] Hollender C, Liu Z (2008). Histone deacetylase genes in *Arabidopsis* development. J. Integr. Plant Biol..

[10] Segal DJ, Dreier B, Beerli RR, Barbas CF (1999). Toward controlling gene expression at will: selection and design of zinc finger domains recognizing each of the 5′-GNN-3′ DNA target sequences. Proc. Natl Acad. Sci. USA.

[11] Johnson LM (2014). SRA- and SET-domain-containing proteins link RNA polymerase V occupancy to DNA methylation. Nature.

[12] Gallego-Bartolome J (2019). Co-targeting RNA polymerases IV and V promotes efficient de novo DNA methylation in *Arabidopsis*. Cell.

[13] Xue Y (2021). *Arabidopsis* MORC proteins function in the efficient establishment of RNA directed DNA methylation. Nat. Commun..

[14] Papikian A, Liu W, Gallego-Bartolome J, Jacobsen SE (2019). Site-specific manipulation of *Arabidopsis* loci using CRISPR–Cas9 SunTag systems. Nat. Commun..

[15] Gardiner J, Ghoshal B, Wang M, Jacobsen SE (2022). CRISPR–CAS mediated transcriptional control and epi-mutagenesis. Plant Physiol..

[16] Tanenbaum ME, Gilbert LA, Qi LS, Weissman JS, Vale RD (2014). A protein-tagging system for signal amplification in gene expression and fluorescence imaging. Cell.

[17] Soppe WJ (2000). The late flowering phenotype of fwa mutants is caused by gain-of-function epigenetic alleles of a homeodomain gene. Mol. Cell.

[18] Yamada K (2003). Empirical analysis of transcriptional activity in the *Arabidopsis* genome. Science.

[19] Pruneda-Paz JL (2014). A genome-scale resource for the functional characterization of *Arabidopsis* transcription factors. Cell Rep..

[20] Guo Y (2021). RAD: a web application to identify region associated differentially expressed genes. Bioinformatics..

[21] He Y, Doyle MR, Amasino RM (2004). PAF1-complex-mediated histone methylation of FLOWERING LOCUS C chromatin is required for the vernalization-responsive, winter-annual habit in *Arabidopsis*. Genes Dev..

[22] Turck F (2007). *Arabidopsis* TFL2/LHP1 specifically associates with genes marked by trimethylation of histone H3 lysine 27. PLoS Genet..

[23] Ueda A (2008). The *Arabidopsis thaliana* carboxyl-terminal domain phosphatase-like 2 regulates plant growth, stress and auxin responses. Plant Mol. Biol..

[24] Liu W (2021). Ectopic targeting of CG DNA methylation in *Arabidopsis* with the bacterial SssI methyltransferase. Nat. Commun..

[25] Derkacheva M (2013). *Arabidopsis* MSI1 connects LHP1 to PRC2 complexes. EMBO J..

[26] Mylne JS (2006). LHP1, the *Arabidopsis* homologue of HETEROCHROMATIN PROTEIN1, is required for epigenetic silencing of FLC. Proc. Natl Acad. Sci. USA.

[27] Zhang X (2007). The *Arabidopsis* LHP1 protein colocalizes with histone H3 Lys27 trimethylation. Nat. Struct. Mol. Biol..

[28] Piunti A, Shilatifard A (2016). Epigenetic balance of gene expression by Polycomb and COMPASS families. Science.

[29] Voigt P, Tee WW, Reinberg D (2013). A double take on bivalent promoters. Genes Dev..

[30] Yang Z (2018). EBS is a bivalent histone reader that regulates floral phase transition in *Arabidopsis*. Nat. Genet..

[31] Lawrence RJ (2004). A concerted DNA methylation/histone methylation switch regulates rRNA gene dosage control and nucleolar dominance. Mol. Cell.

[32] Chen X (2018). Canonical and noncanonical actions of *Arabidopsis* histone deacetylases in ribosomal RNA processing. Plant Cell.

[33] To TK (2011). Arabidopsis HDA6 regulates locus-directed heterochromatin silencing in cooperation with MET1. PLoS Genet..

[34] Lin J (2020). HDA6-dependent histone deacetylation regulates mRNA polyadenylation in *Arabidopsis*. Genome Res..

[35] Earley K (2006). Erasure of histone acetylation by *Arabidopsis* HDA6 mediates large-scale gene silencing in nucleolar dominance. Genes Dev..

[36] Xu Y (2022). MSI1 and HDA6 function interdependently to control flowering time via chromatin modifications. Plant J..

[37] Mehdi S (2016). The WD40 domain protein MSI1 functions in a histone deacetylase complex to fine-tune abscisic acid signaling. Plant Cell.

[38] Fukuda H, Sano N, Muto S, Horikoshi M (2006). Simple histone acetylation plays a complex role in the regulation of gene expression. Brief. Funct. Genom. Proteomic.

[39] Tomson BN, Arndt KM (2013). The many roles of the conserved eukaryotic Paf1 complex in regulating transcription, histone modifications, and disease states. Biochim. Biophys. Acta.

[40] Antosz W (2017). The composition of the *Arabidopsis* RNA polymerase II transcript elongation complex reveals the interplay between elongation and mRNA processing factors. Plant Cell.

[41] Oh S, Park S, van Nocker S (2008). Genic and global functions for Paf1C in chromatin modification and gene expression in *Arabidopsis*. PLoS Genet..

[42] Alonso JM (2003). Genome-wide insertional mutagenesis of *Arabidopsis thaliana*. Science.

[43] Hou L (2019). Paf1C regulates RNA polymerase II progression by modulating elongation rate. Proc. Natl Acad. Sci. USA.

[44] Fischl H, Howe FS, Furger A, Mellor J (2017). Paf1 has distinct roles in transcription elongation and differential transcript fate. Mol. Cell.

[45] Koiwa H (2004). *Arabidopsis* C-terminal domain phosphatase-like 1 and 2 are essential Ser-5-specific C-terminal domain phosphatases. Proc. Natl Acad. Sci. USA.

[46] Zhang YZ (2020). Coupling of H3K27me3 recognition with transcriptional repression through the BAH-PHD-CPL2 complex in *Arabidopsis*. Nat. Commun..

[47] Qian F (2021). A histone H3K27me3 reader cooperates with a family of PHD finger-containing proteins to regulate flowering time in *Arabidopsis*. J. Integr. Plant Biol..

[48] Dalmay T, Hamilton A, Rudd S, Angell S, Baulcombe DC (2000). An RNA-dependent RNA polymerase gene in *Arabidopsis* is required for posttranscriptional gene silencing mediated by a transgene but not by a virus. Cell.

[49] Luo M (2012). HD2C interacts with HDA6 and is involved in ABA and salt stress response in *Arabidopsis*. J. Exp. Bot..

[50] Xi Y, Li W (2009). BSMAP: whole genome bisulfite sequence MAPping program. BMC Bioinform..

[51] Langmead B, Salzberg SL (2012). Fast gapped-read alignment with Bowtie 2. Nat. Methods.

[52] Li H (2009). The Sequence Alignment/Map format and SAMtools. Bioinformatics.

[53] Ramirez F (2016). deepTools2: a next generation web server for deep-sequencing data analysis. Nucleic Acids Res..

[54] Zhang Y (2008). Model-based analysis of ChIP–seq (MACS). Genome Biol..

[55] Li B, Dewey CN (2011). RSEM: accurate transcript quantification from RNA-seq data with or without a reference genome. BMC Bioinform..

[56] Grabherr MG (2011). Full-length transcriptome assembly from RNA-seq data without a reference genome. Nat. Biotechnol..

