# Supplementary material for: A gene silencing screen uncovers diverse tools for targeted gene repression in Arabidopsis
Source: Nat Plants. 2023 Mar 6;9(3):460–72. doi: 10.1038/s41477-023-01362-8 (PMC10027610; doi:10.1038/s41477-023-01362-8)
Supplement: Supplementary file 1 — Supplementary figs. 1–6. [file 41477_2023_1362_MOESM1_ESM.pdf]

nature plants

Article

https://doi.org/10.1038/s41477-023-01362-8

A gene silencing screen uncovers diverse tools for targeted gene repression in *Arabidopsis*

In the format provided by the authors and unedited

Table-of-contents

Supplementary Fig. **1** to **6**.

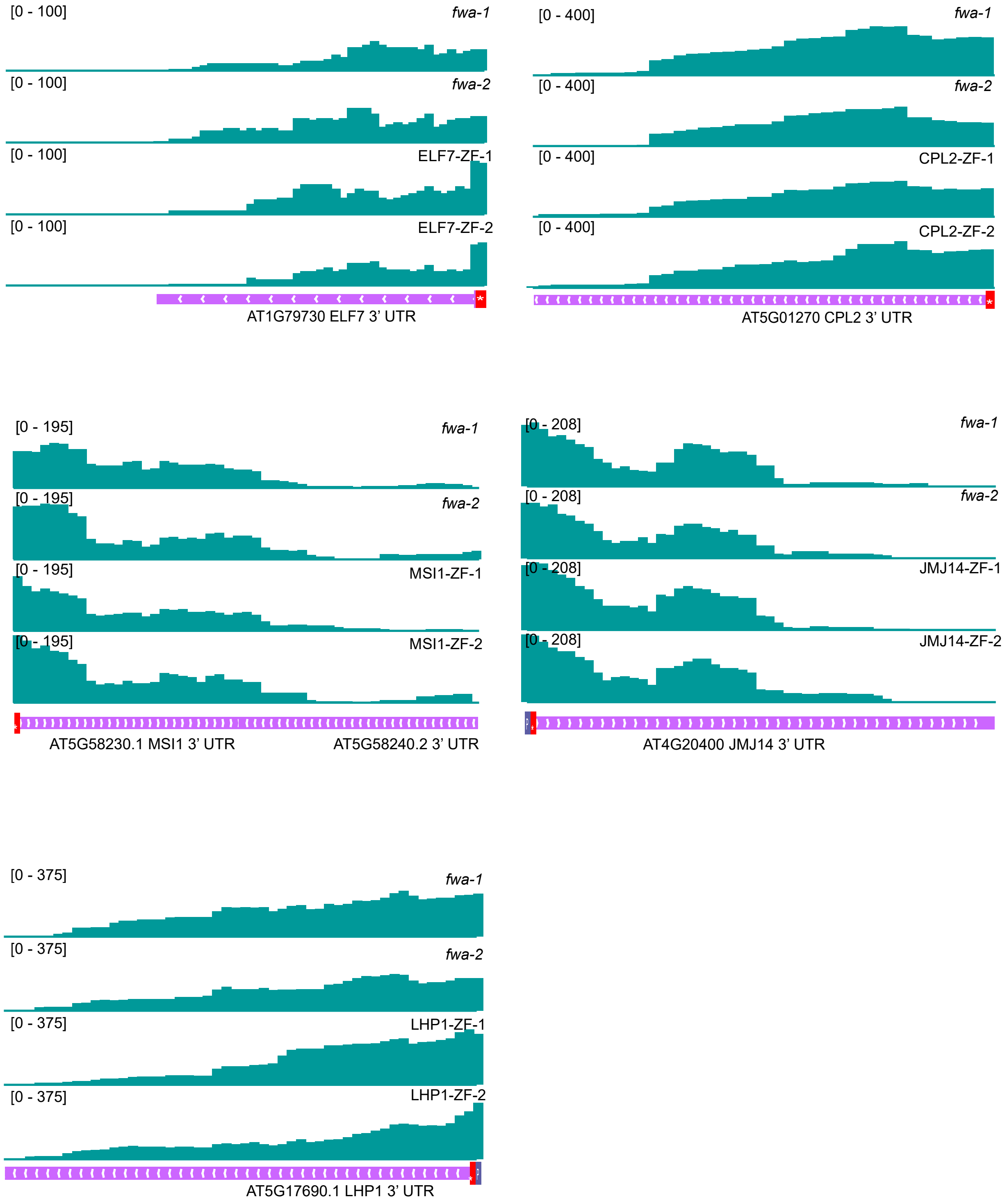

Supplementary Fig. 1: The endogenous genes were not silenced in the respective ZF transgenic lines. Screen shots showing the RNA-seq of endogenous gene expression level (3' UTR) in *fwa* and ZF lines.

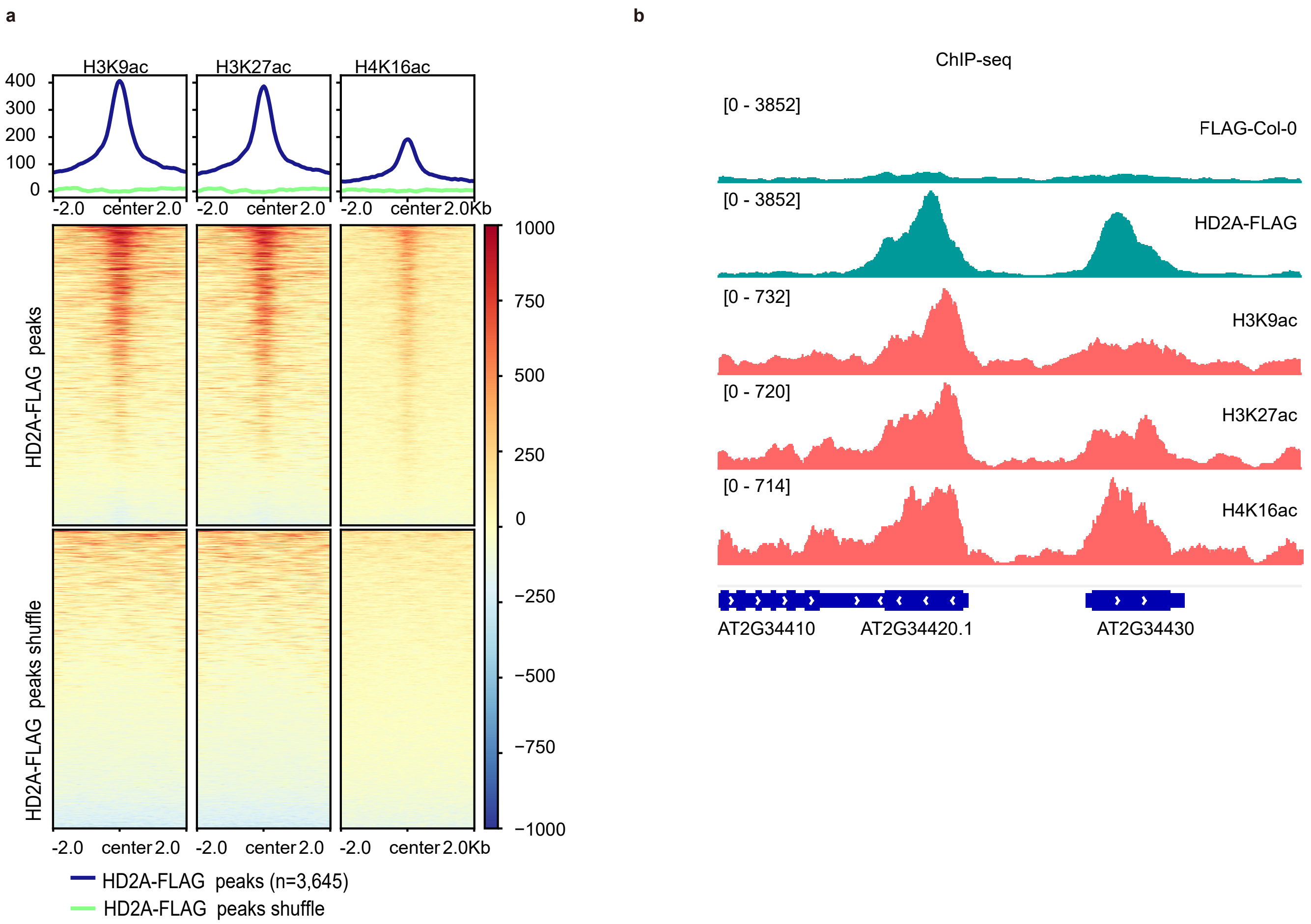

Supplementary Fig. 2: HD2A overlapped with histone H3K9ac, H3K27ac, and H4K16ac. a, Heatmaps and metaplots representing the H3K9ac, H3K27ac, and H4K16ac ChIP-seq signals over HD2A-FLAG ChIP-seq peaks (n=3,645, top panel) and shuffle sites (bottom panel). **b,** Screenshots showing the FLAG ChIP-seq signals in Col-0, HD2A-FLAG, and histone H3K9ac, H3K27ac, and H4K16ac ChIP-seq signals in Col-0 over two representative genes.

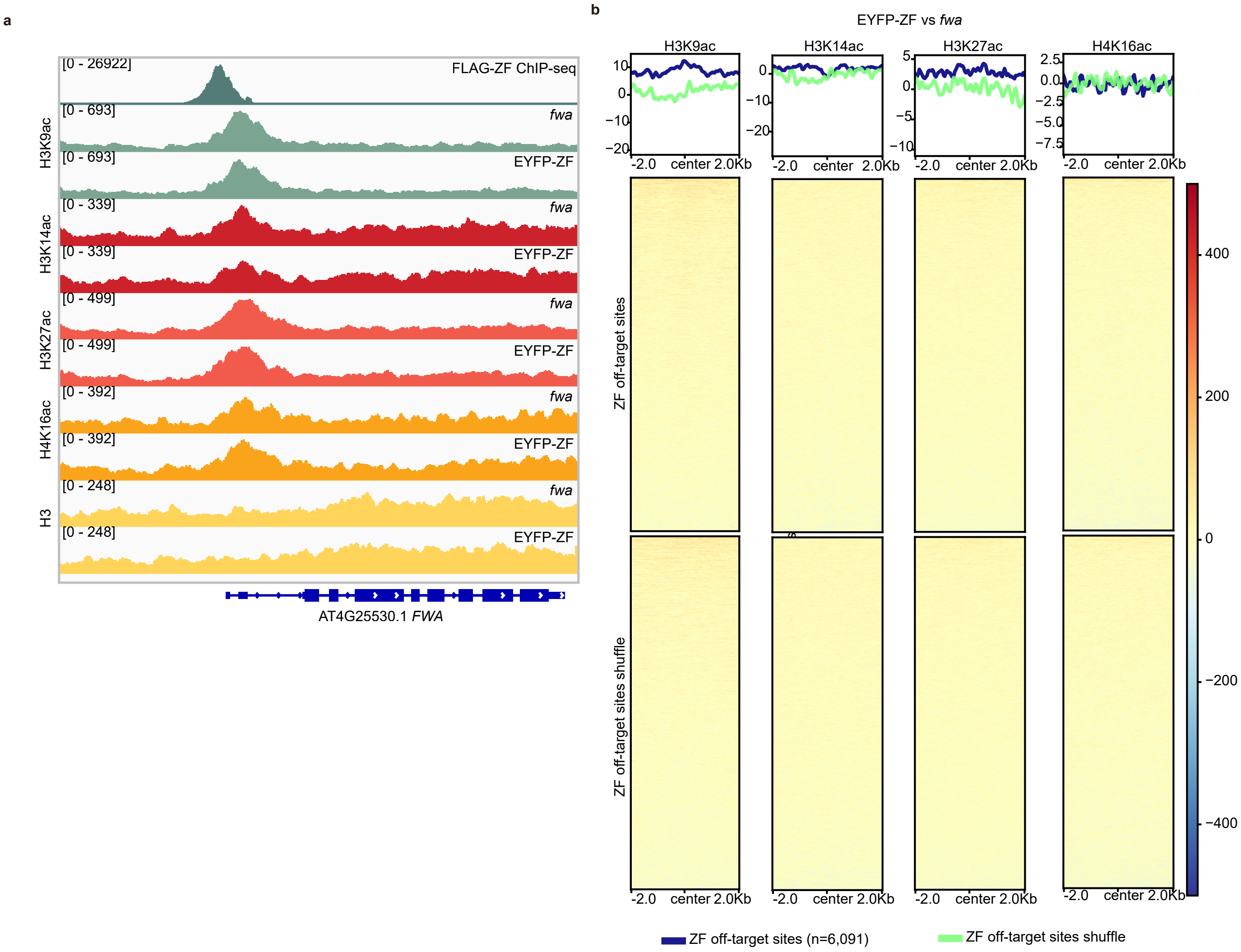

Supplementary Fig. 3: Histone acetylation levels were not reduced in EYFP-ZF T2 transgenic lines. a, Screenshots indicating H3K9ac, H3K14ac, H3K27ac, H4K16ac, and H3 ChIP-seq signals over *FWA* locus in *fwa* and T2 transgenic lines of EYFP-ZF. **b,** Heatmaps and metaplots depicting normalized H3K9ac, H3K14ac, H3K27ac, H4K16ac ChIP-seq signals over ZF off-target sites (n=6,091) and shuffle sites in EYFP-ZF transgenic lines versus *fwa*.

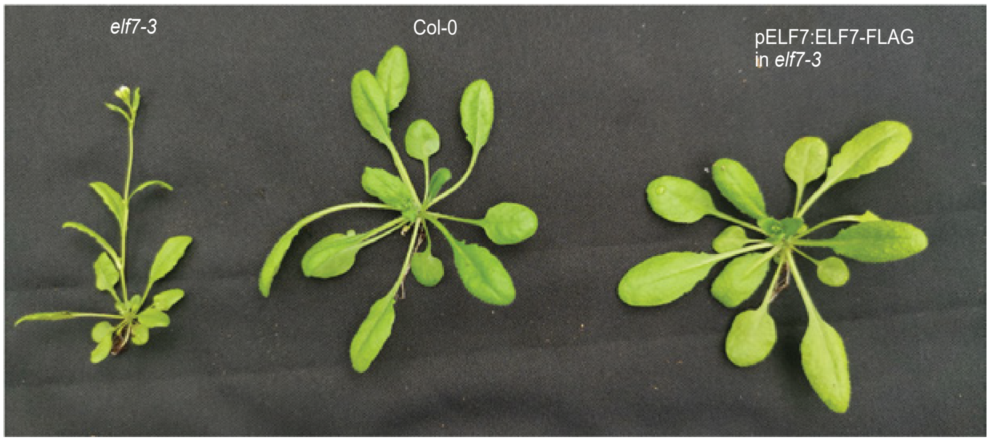

Supplementary Fig. 4: *elf7-3* mutant was successfully complemented by pELF7:ELF7-FLAG. Phenotype of 3-4-week-old Arabidopsis *elf7-3* mutant, Col-0 wild type, and pELF7:ELF7-FLAG transgenic line in the background of *elf7-3*.

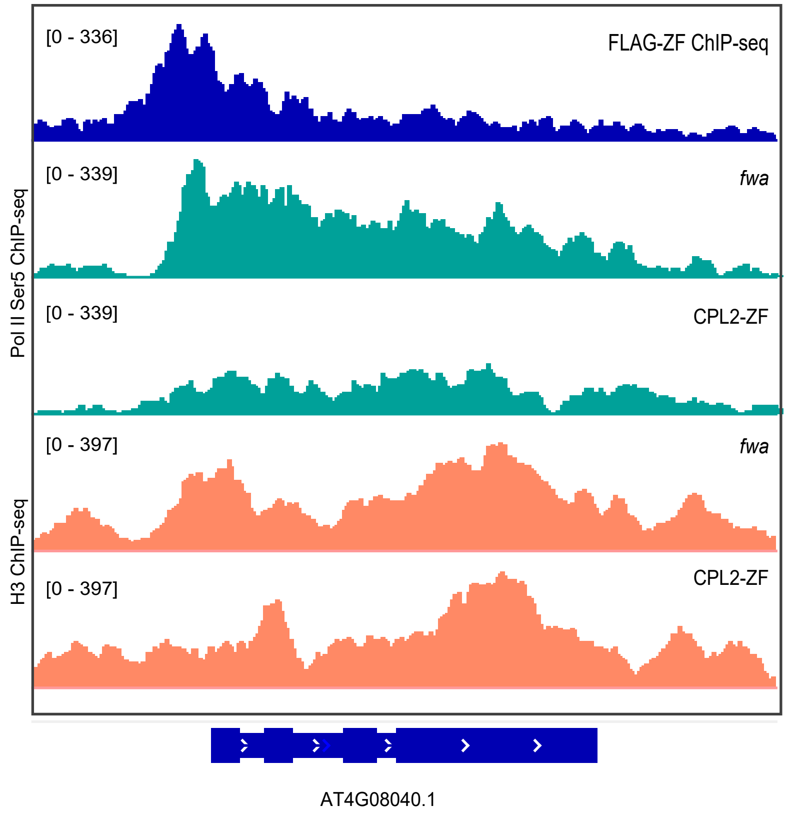

Supplementary Fig. 5: Target gene silencing by CPL2-ZF and pol II Ser5 dephosphorylation. Screenshots showing FLAG-ZF and Pol II ChIP-seq signals in *fwa* and T2 transgenic line of CPL2-ZF over a representative ZF off-target site.

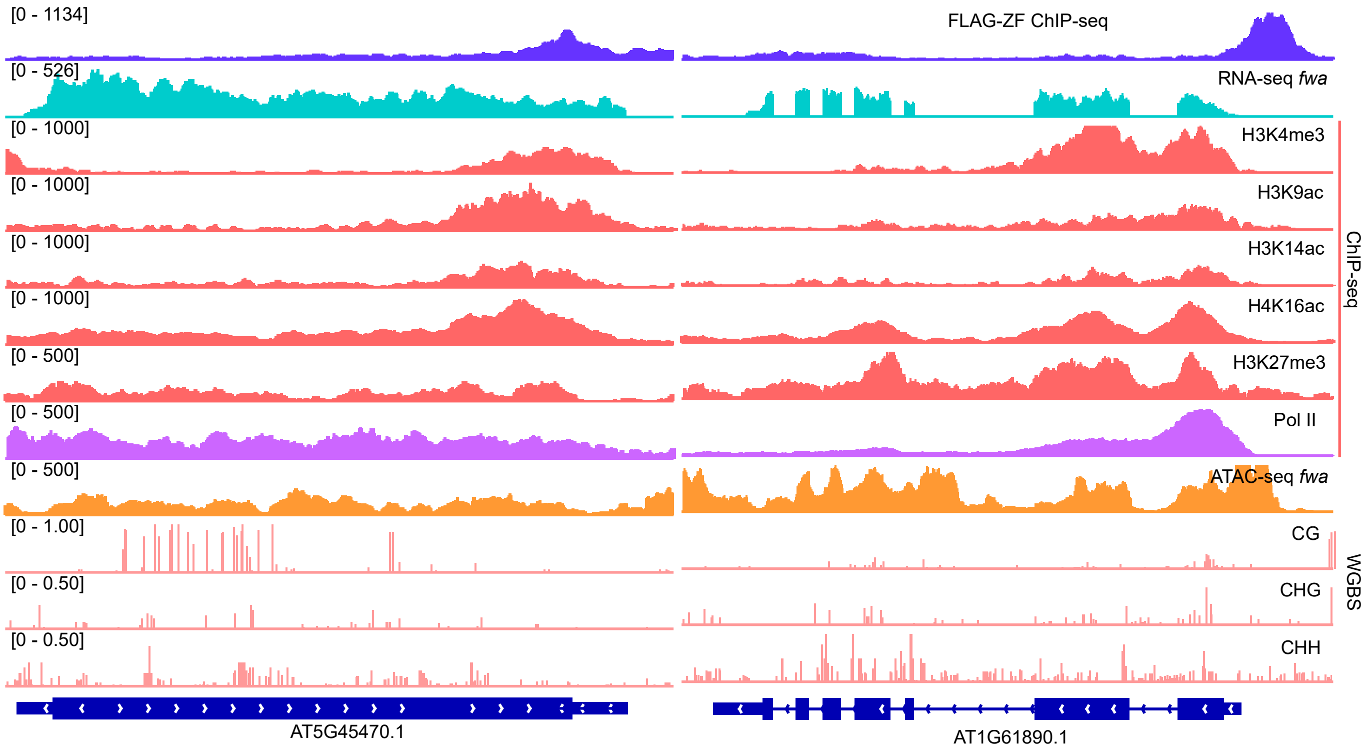

Supplementary Fig. 6: ZF target genes associated chromatin features are varied. Screenshots of FLAG-ZF ChIP-seq and RNA-seq in *fwa*, ChIP-seq signals of H3K4me3, H3K9ac, H3K14ac, H4K16ac, H3K27me3, and Pol II in *fwa*, ATAC-seq signals in *fwa*, and CG, CHG, and CHH DNA methylation levels in *fwa* over two representative ZF targeting genes.
