# Supplementary material for: A gene silencing screen uncovers diverse tools for targeted gene repression in Arabidopsis
Source: Nat Plants. 2023 Mar 6;9(3):460–72. doi: 10.1038/s41477-023-01362-8 (PMC10027610; doi:10.1038/s41477-023-01362-8)
Supplement: Supplementary file 2 — Reporting Summary [file 41477_2023_1362_MOESM2_ESM.pdf]

nature portfolio

Corresponding author(s): Steve Jacobsen

Last updated by author(s): Jan 18, 2023

Reporting Summary

Nature Portfolio wishes to improve the reproducibility of the work that we publish. This form provides structure for consistency and transparency in reporting. For further information on Nature Portfolio policies, see our Editorial Policies and the Editorial Policy Checklist.

Statistics

For all statistical analyses, confirm that the following items are present in the figure legend, table legend, main text, or Methods section.

n/a	Confirmed	
☐	☒	The exact sample size (*n*) for each experimental group/condition, given as a discrete number and unit of measurement
☒	☐	A statement on whether measurements were taken from distinct samples or whether the same sample was measured repeatedly
☐	☒	The statistical test(s) used AND whether they are one- or two-sided<br>*Only common tests should be described solely by name; describe more complex techniques in the Methods section.*
☒	☐	A description of all covariates tested
☒	☐	A description of any assumptions or corrections, such as tests of normality and adjustment for multiple comparisons
☐	☒	A full description of the statistical parameters including central tendency (e.g. means) or other basic estimates (e.g. regression coefficient) AND variation (e.g. standard deviation) or associated estimates of uncertainty (e.g. confidence intervals)
☐	☒	For null hypothesis testing, the test statistic (e.g. *F*, *t*, *r*) with confidence intervals, effect sizes, degrees of freedom and *P* value noted<br>*Give P values as exact values whenever suitable.*
☒	☐	For Bayesian analysis, information on the choice of priors and Markov chain Monte Carlo settings
☒	☐	For hierarchical and complex designs, identification of the appropriate level for tests and full reporting of outcomes
☒	☐	Estimates of effect sizes (e.g. Cohen's *d*, Pearson's *r*), indicating how they were calculated

Our web collection on statistics for biologists contains articles on many of the points above.

Software and code

Policy information about availability of computer code

Data collection

No software was used for data collection.

Data analysis

RNA-seq analysis
Cleaned short reads were aligned to reference genome tair10 by Bowtie2 (v2.1.0), and expression abundance was calculated by RSEM (v1.3.1) with default parameters. Heatmaps were visualized with the R package pheatmap (v1.0.12). To reduce false positive of differential expression, transcripts with less than 5 reads of all replicates in total were regarded as lowly expressed genes and have been removed in subsequent analysis. Differential expression analysis was conducted using edgeR (v3.32.1). A threshold of p value < 0.05 and Fold Change > 2 were used to decide whether significant expression difference exists between samples.

ChIP-seq analysis
ChIP-seq fastq reads were aligned to the TAIR10 reference genome with Bowtie2 (v2.1.0), allowing only uniquely mapping reads with 0 mismatches. Duplicated reads were removed by Samtools. ChIP-seq peaks were called by MACS2 (v2.1.1) and annotated with ChIPseeker (v1.28.3). Differential peaks were called by bdgdiff function in MACS2. ChIP-seq data metaplots were plotted by deeptools (v2.5.1). For Pol II 5' occupancy analysis, Pol II occupancy was calculated based normalized reads count (RPKM) on a TSS +/- 200 bp region and a TSS +500 bp to TTS gene body region by bedtools. Detailed information for published ChIP-seq datasets is listed in Supplementary Table 2.

Trim_galore (http://www.bioinformatics.babraham.ac.uk/projects/trim_galore/) was used to trim adapters after filtering low quality reads. BS-seq reads were aligned to TAIR10 reference genome by Bismark (v0.18.2) with default settings. Reads with three or more consecutive CHH sites were considered as unconverted reads and filtered. DNA methylation levels were defined as #C/ (#C + #T). DMRs (Differentially Methylated Regions) were called by DMRcaller with p < 0.01 for where the differences in CG, CHG, and CHH methylation were at least 0.4, 0.2, and 0.1, respectively.

BS-PCR analysis
BS-PCR data were trimmed with primer sequences and mapped to TAIR10 reference genome with bsmap (v2.90) allowing 2 mismatches and 1 best hit (-v 2 -w 1).

For manuscripts utilizing custom algorithms or software that are central to the research but not yet described in published literature, software must be made available to editors and reviewers. We strongly encourage code deposition in a community repository (e.g. GitHub). See the Nature Portfolio guidelines for submitting code & software for further information.

Data

Policy information about availability of data

All manuscripts must include a data availability statement. This statement should provide the following information, where applicable:

- Accession codes, unique identifiers, or web links for publicly available datasets
- A description of any restrictions on data availability
- For clinical datasets or third party data, please ensure that the statement adheres to our policy

All high-throughput sequencing data generated in this study are accessible at NCBI's Gene Expression Omnibus (GEO) via GEO Series accession number GSE197063(https://www.ncbi.nlm.nih.gov/geo/query/acc.cgi?acc=GSE197063). Tair10 genome is available at https://www.arabidopsis.org/index.jsp

Field-specific reporting

Please select the one below that is the best fit for your research. If you are not sure, read the appropriate sections before making your selection.

☒ Life sciences ☐ Behavioural & social sciences ☐ Ecological, evolutionary & environmental sciences

For a reference copy of the document with all sections, see nature.com/documents/nr-reporting-summary-flat.pdf

Life sciences study design

All studies must disclose on these points even when the disclosure is negative.

Sample size	No sample size calculation was performed. The applied sample sizes for RNA-seq, WGBS, ChIP-seq, and BS-PCR etc al, were selected according to public standards in the field.
Data exclusions	No data exclusion in the study.
Replication	Two replicates for ChIP-seq. Two replicates for BS-PCR. Three replicates for RNA-seq samples. Three technical replicates for qRT-PCR. All replicates were performed independently and produced high reproducible results.
Randomization	For all experiments, treatment and control samples were grown side by side, each replicate on separate plate. Allocation of samples were not random, because it is not relevant to the study.
Blinding	No blinding used because it was largely not relevant to our study. All data were collected based on the genotype of plants, while blinding the samples during the experiments will increases the risk of mislabeling and wrong results.

Reporting for specific materials, systems and methods

We require information from authors about some types of materials, experimental systems and methods used in many studies. Here, indicate whether each material, system or method listed is relevant to your study. If you are not sure if a list item applies to your research, read the appropriate section before selecting a response.

Materials & experimental systems

n/a	Involved in the study	
☐	☒	Antibodies
☒	☐	Eukaryotic cell lines
☒	☐	Palaeontology and archaeology
☒	☐	Animals and other organisms
☒	☐	Human research participants
☒	☐	Clinical data
☒	☐	Dual use research of concern

Methods

n/a	Involved in the study	
☐	☒	ChIP-seq
☒	☐	Flow cytometry
☒	☐	MRI-based neuroimaging

Antibodies

Antibodies used	Anti-FLAG Millipore Sigma Cat# F1804; RRID:AB_262044

Antibodies used

Anti-FLAG M2-Peroxidase (HRP) Millipore Sigma Sigma-Aldrich Cat# A8592, RRID:AB_439702
Anti-FLAG® M2 Magnetic Beads Millipore Sigma Cat# M8823, RRID:AB_2637089
Anti-Histone H3 Abcam Cat# ab1791, RRID:AB_302613
Anti-trimethyl-Histone H3 (Lys4)¬ Millipore Sigma Cat# 04-745, RRID:AB_1163444
Anti-Histone H3 (acetyl K9) Abcam Cat# ab4441, RRID:AB_2118292
Anti-Histone H3 (acetyl K14) Abcam Cat# ab52946, RRID:AB_880442
Anti-Histone H3 (acetyl K27) Abcam Cat# ab4729, RRID:AB_2118291
Anti-trimethyl-Histone H3 (Lys27) Millipore Sigma Cat# 07-449, RRID:AB_310624
Anti-Histone H3 (di methyl K36) Abcam Cat# ab9049, RRID:AB_1280939
Anti-Histone H3 (tri methyl K36) Abcam Cat# ab9050, RRID:AB_306966
Anti-Histone H4 (acetyl K16) Abcam Cat# ab109463, RRID:AB_10858987
Anti-RNA polymerase II CTD repeat YSPTSPS (phospho S5) antibody Abcam Cat# ab5131, RRID:AB_449369

Validation

anti-FLAG M2 (Sigma): the antibodies have been validated by the manufacturer, https://www.sigmaaldrich.com/catalog/product/sigma/fl804
Anti-FLAG M2-Peroxidase (HRP)(Sigma): the antibodies have been validated by the manufacturer, https://www.sigmaaldrich.com/US/en/product/sigma/a8592
Anti-Pol II Ser 5 (Abeam ab5131): the antibodies have been validated by the manufacturer, https://www.abcam.com/rna-polymerase-ii-ctd-repeat-ysptsps-phospho-s5-antibody-ab5131.html
anti-H3 (Ab1791, Abeam): the antibodies have been validated by the manufacturer, https://www.abcam.com/histone-h3-antibody-nuclear-marker-and-chip-grade-ab1791.html
anti-H3K36me2 (Ab9049, Abeam): the antibodies have been validated by the manufacturer, https://www.abcam.com/histone-h3-di-methyl-k36-antibody-chip-grade-ab9049.html
anti-H3K36me3 (Ab9050, Abeam): the antibodies have been validated by the manufacturer, https://www.abcam.com/histone-h3-tri-methyl-k36-antibody-ch ip-grade-ab9050.html
Anti-H3K27me3 (07-449, Millipore Sigma): the antibodies have been validated by the manufacturer, https://www.emdmiliipore.com/US/en/product/ Anti-trimethyl-Histone-H3-Lys27-Antibody,MM_NF-07-449
Anti-H3K4me3 (04-745, Millipore Sigma): the antibodies have been validated by the manufacturer, https://www.emdmillipore.com/US/en/product/Anti-trimethyl-Histone-H3-Lys4-Antibodyclone-MC315-rabbit-monoclona I, MM_NF-04-745
Anti-H3K27ac (ab4729, Abcam) : the antibodies have been validated by the manufacturer, https://www.abcam.com/histone-h3-acetyl-k27-antibody-chip-grade-ab4729.html
Anti-H3K9ac (ab4441, Ab cam) :the antibodies have been validated by the manufacturer, https://www.abcam.com/h istone-h3-acetyl-k9-a ntibody-ch ip-grade-ab4441.html
Anti-H3K14ac (ab82501, abcam): the antibodies have been validated by the manufacturer, https ://www.abcam.com/histone-h3-acetyl-k14-antibody-ab82501.html

Data deposition

☒ Confirm that both raw and final processed data have been deposited in a public database such as GEO.

☒ Confirm that you have deposited or provided access to graph files (e.g. BED files) for the called peaks.

Data access links
May remain private before publication.

All high-throughput sequencing data generated in this study are accessible at NCBI's Gene Expression Omnibus (GEO) via GEO Series accession number GSE197063(https://www.ncbi.nlm.nih.gov/geo/query/acc.cgi?acc=GSE197063). Enter token qvetmgogbnqxxmp into the box.

Files in database submission

ChIPseq-SET1-H3-ELF7-ZF-rep1.bw
ChIPseq-SET1-H3-fwa-rep1.bw
ChIPseq-SET1-H3-JMJ14-ZF-rep1.bw
ChIPseq-SET1-H3-LHP1-ZF-rep1.bw
ChIPseq-SET1-H3-MSI1-ZF-rep1.bw
ChIPseq-SET1-H3K27me3-ELF7-ZF-rep1.bw
ChIPseq-SET1-H3K27me3-fwa-rep1.bw
ChIPseq-SET1-H3K27me3-JMJ14-ZF-rep1.bw
ChIPseq-SET1-H3K27me3-LHP1-ZF-rep1.bw
ChIPseq-SET1-H3K27me3-MSI1-ZF-rep1.bw
ChIPseq-SET1-H3K4me3-ELF7-ZF-rep1.bw
ChIPseq-SET1-H3K4me3-fwa-rep1.bw
ChIPseq-SET1-H3K4me3-JMJ14-ZF-rep1.bw
ChIPseq-SET10-H3-CPL2-ZF-rep1.bw
ChIPseq-SET10-H3-fwa-rep1.bw
ChIPseq-SET10-PolII-CPL2-ZF-rep1.bw
ChIPseq-SET10-PolII-fwa-rep1.bw
ChIPseq-SET11-H3-CPL2-ZF-rep2.bw
ChIPseq-SET11-H3-fwa-rep2.bw
ChIPseq-SET11-PolII-CPL2-ZF-rep2.bw
ChIPseq-SET11-PolII-fwa-rep2.bw
ChIPseq-SET12-H3-Col-0-rep1.bw
ChIPseq-SET12-H3-elf7-rep1.bw
ChIPseq-SET12-PolII-Col-0-rep1.bw
ChIPseq-SET12-PolII-elf7-rep1.bw
ChIPseq-SET13-H3-Col-0-rep2.bw

ChIPseq-SET13-H3-elf7-rep2.bw
ChIPseq-SET13-PolII-Col-0-rep2.bw
ChIPseq-SET13-PolII-elf7-rep2.bw
ChIPseq-SET14-H3-ELF7-ZF-rep1.bw
ChIPseq-SET14-H3-EYFP-ZF-rep1_S3_L003.bw
ChIPseq-SET14-H3-EYFP-ZF-rep2_S4_L003.bw
ChIPseq-SET14-H3-fwa-rep1_S1_L003.bw
ChIPseq-SET14-H3-fwa-rep2_S2_L003.bw
ChIPseq-SET14-H3K27me3-EYFP-ZF-rep1.bw
ChIPseq-SET14-H3K27me3-EYFP-ZF-rep2.bw
ChIPseq-SET14-H3K27me3-fwa-rep1_S14_L003.bw
ChIPseq-SET14-H3K27me3-fwa-rep2.bw
ChIPseq-SET14-H3K36me2-ELF7-ZF-rep1.bw
ChIPseq-SET14-H3K36me2-fwa-rep1_S25_L003.bw
ChIPseq-SET14-H3K36me3-ELF7-ZF-rep1.bw
ChIPseq-SET14-H3K36me3-fwa-rep1.bw
ChIPseq-SET14-H3K4me3-EYFP-ZF-rep1_S11_L003.bw
ChIPseq-SET14-H3K4me3-EYFP-ZF-rep2.bw
ChIPseq-SET14-H3K4me3-fwa-rep1_S9_L003.bw
ChIPseq-SET14-H3K4me3-fwa-rep2_S10_L003.bw
ChIPseq-SET14-PolII-EYFP-ZF-rep1_S21_L003.bw
ChIPseq-SET14-PolII-EYFP-ZF-rep2_S22_L003.bw
ChIPseq-SET14-PolII-fwa-rep1.bw
ChIPseq-SET14-PolII-fwa-rep2.bw
ChIPseq-SET15-H3-EYFP-ZF-rep1.bw
ChIPseq-SET15-H3-EYFP-ZF-rep2.bw
ChIPseq-SET15-H3-fwa-rep1.bw
ChIPseq-SET15-H3-fwa-rep2.bw
ChIPseq-SET15-H3K14ac-EYFP-ZF-rep1.bw
ChIPseq-SET15-H3K14ac-EYFP-ZF-rep2_S58_L003.bw
ChIPseq-SET15-H3K14ac-fwa-rep1_S55_L003.bw
ChIPseq-SET15-H3K14ac-fwa-rep2.bw
ChIPseq-SET15-H3K27ac-EYFP-ZF-rep1.bw
ChIPseq-SET15-H3K27ac-EYFP-ZF-rep2.bw
ChIPseq-SET15-H3K27ac-fwa-rep1.bw
ChIPseq-SET15-H3K27ac-fwa-rep2_S46_L003.bw
ChIPseq-SET15-H3K9ac-EYFP-ZF-rep1_S41_L003.bw
ChIPseq-SET15-H3K9ac-EYFP-ZF-rep2.bw
ChIPseq-SET15-H3K9ac-fwa-rep1.bw
ChIPseq-SET15-H3K9ac-fwa-rep2_S40_L003.bw
ChIPseq-SET15-H4K16ac-EYFP-ZF-rep1.bw
ChIPseq-SET15-H4K16ac-EYFP-ZF-rep2.bw
ChIPseq-SET15-H4K16ac-fwa-rep1.bw
ChIPseq-SET15-H4K16ac-fwa-rep2_S51_L003.bw
ChIPseq-SET16-FLAG-Col-0-rep1.bw
ChIPseq-SET16-FLAG-ELF7-rep1.bw
ChIPseq-SET16-FLAG-fwa.bw
ChIPseq-SET16-FLAG-TRB1-ZF.bw
ChIPseq-SET17-FLAG-Col-0-rep2.bw
ChIPseq-SET17-FLAG-ELF7-rep2.bw
ChIPseq-SET18-FLAG-Col-0-rep1.bw
ChIPseq-SET18-FLAG-HD2A-rep1.bw
ChIPseq-SET2-H3-ELF7-ZF-rep2.bw
ChIPseq-SET2-H3-fwa-rep2.bw
ChIPseq-SET2-H3-JMJ14-ZF-rep2.bw
ChIPseq-SET2-H3-LHP1-ZF-rep2.bw
ChIPseq-SET2-H3-MSI1-ZF-rep2.bw
ChIPseq-SET2-H3K27me3-ELF7-ZF-rep2.bw
ChIPseq-SET2-H3K27me3-fwa-rep2.bw
ChIPseq-SET2-H3K27me3-JMJ14-ZF-rep2.bw
ChIPseq-SET2-H3K27me3-LHP1-ZF-rep2.bw
ChIPseq-SET2-H3K27me3-MSI1-ZF-rep2.bw
ChIPseq-SET2-H3K4me3-ELF7-ZF-rep2.bw
ChIPseq-SET2-H3K4me3-fwa-rep2.bw
ChIPseq-SET2-H3K4me3-JMJ14-ZF-rep2.bw
ChIPseq-SET20-H3-ELF7-ZF-rep2.bw
ChIPseq-SET20-H3-fwa-rep2.bw
ChIPseq-SET20-H3K36me2-ELF7-ZF-rep2.bw
ChIPseq-SET20-H3K36me2-fwa-rep2.bw
ChIPseq-SET20-H3K36me3-ELF7-ZF-rep2.bw
ChIPseq-SET20-H3K36me3-fwa-rep2.bw
ChIPseq-SET3-H3-fwa1-rep1.bw
ChIPseq-SET3-H3-fwa2-rep1.bw
ChIPseq-SET3-H3-HD2A-ZF-rep1.bw
ChIPseq-SET3-H3-HD2B-ZF-rep1.bw
ChIPseq-SET3-H3-HD2C-ZF-rep1.bw

Methodology

Replicates

2

Sequencing depth

Name Total_reads Unique_reads Reads_length Reads_type
ChIPseq-SET1-H3-ELF7-ZF-rep1_S20_L003 34692333 30138805 50 PE
ChIPseq-SET1-H3-fwa-rep1_S22_L003 34615984 29849162 50 PE
ChIPseq-SET1-H3-JMJ14-ZF-rep1_S35_L003 30624027 24609533 50 PE
ChIPseq-SET1-H3-LHP1-ZF-rep1_S16_L003 36532278 31760176 50 PE
ChIPseq-SET1-H3-MSI1-ZF-rep1_S19_L003 34773551 29846276 50 PE
ChIPseq-SET1-H3K27me3-ELF7-ZF-rep1_S85_L003 16857198 13565042 50 PE
ChIPseq-SET1-H3K27me3-fwa-rep1_S48_L003 24850552 19542262 50 PE
ChIPseq-SET1-H3K27me3-JMJ14-ZF-rep1_S91_L003 13614616 9023791 50 PE
ChIPseq-SET1-H3K27me3-LHP1-ZF-rep1_S70_L003 19171383 14191867 50 PE
ChIPseq-SET1-H3K27me3-MSI1-ZF-rep1_S47_L003 25550724 19876895 50 PE
ChIPseq-SET1-H3K4me3-ELF7_-ZF-rep1 17892582 14665344 50 PE
ChIPseq-SET1-H3K4me3-fwa-rep1 26780511 22388582 50 PE
ChIPseq-SET1-H3K4me3-JMJ14-ZF-rep1 16201931 12494941 50 PE
ChIPseq-SET10-H3-CPL2-ZF-rep1 45106998 40540319 50 PE
ChIPseq-SET10-H3-fwa-rep1 54359212 48847610 50 PE
ChIPseq-SET10-PolII-CPL2-ZF-rep1 33785877 28604913 50 PE
ChIPseq-SET10-PolII-fwa-rep1 32512367 28918814 50 PE
ChIPseq-SET11-H3-CPL2-ZF-rep2 30282941 27328876 50 PE
ChIPseq-SET11-H3-fwa-rep2 34762270 31665246 50 PE
ChIPseq-SET11-PolII-CPL2-ZF-rep2 33302491 28278239 50 PE
ChIPseq-SET11-PolII-fwa-rep2 29869149 24921260 50 PE
ChIPseq-SET12-H3-Col-0-rep1 36471469 30832953 50 PE
ChIPseq-SET12-H3-elf7-rep1 30482761 26831959 50 PE
ChIPseq-SET12-PolII-Col-0-rep1 21776017 18795866 50 PE
ChIPseq-SET12-PolII-elf7-rep1 21635631 18731688 50 PE
ChIPseq-SET13-H3-Col-0-rep2 45824394 41912020 50 PE
ChIPseq-SET13-H3-elf7-rep2 37223321 33652002 50 PE
ChIPseq-SET13-PolII-Col-0-rep2 27287358 24294860 50 PE
ChIPseq-SET13-PolII-elf7-rep2 19988675 17608335 50 PE
ChIPseq-SET14-H3-ELF7-ZF-rep1 65351053 53822838 50 PE
ChIPseq-SET14-H3-EYFP-ZF-rep1_S3_L003 60530650 44916068 50 PE
ChIPseq-SET14-H3-EYFP-ZF-rep2_S4_L003 59588234 44961384 50 PE
ChIPseq-SET14-H3-fwa-rep1 72788551 57831743 50 PE
ChIPseq-SET14-H3-fwa-rep2 57686916 48104153 50 PE
ChIPseq-SET14-H3K27me3-EYFP-ZF-rep1_S16_L003 31084636 17847186 50 PE
ChIPseq-SET14-H3K27me3-EYFP-ZF-rep2 40418814 18323190 50 PE
ChIPseq-SET14-H3K27me3-fwa-rep1 40315526 18877298 50 PE
ChIPseq-SET14-H3K27me3-fwa-rep2 35664069 24163516 50 PE
ChIPseq-SET14-H3K36me2-ELF7-ZF-rep1 32259193 23676068 50 PE
ChIPseq-SET14-H3K36me2-fwa-rep1_S25_L003 30263355 22711628 50 PE
ChIPseq-SET14-H3K36me3-ELF7-ZF-rep1 51708205 39152947 50 PE
ChIPseq-SET14-H3K36me3-fwa-rep1 56600189 39809288 50 PE
ChIPseq-SET14-H3K4me3-EYFP-ZF-rep1 36933595 22418852 50 PE
ChIPseq-SET14-H3K4me3-EYFP-ZF-rep2 36490756 23447340 50 PE
ChIPseq-SET14-H3K4me3-fwa-rep1 41475420 28572652 50 PE
ChIPseq-SET14-H3K4me3-fwa-rep2 27195069 19783991 50 PE
ChIPseq-SET14-PolII-EYFP-ZF-rep1 29680365 20620976 50 PE
ChIPseq-SET14-PolII-EYFP-ZF-rep2 44560864 31033634 50 PE
ChIPseq-SET14-PolII-fwa-rep1 30298421 22873363 50 PE
ChIPseq-SET14-PolII-fwa-rep2 28536886 21535184 50 PE
ChIPseq-SET15-H3-EYFP-ZF-rep1 52324342 41816662 50 PE
ChIPseq-SET15-H3-EYFP-ZF-rep2 44051707 35836694 50 PE
ChIPseq-SET15-H3-fwa-rep1 76132941 64435746 50 PE
ChIPseq-SET15-H3-fwa-rep2 53395517 41430947 50 PE
ChIPseq-SET15-H3K14ac-EYFP-ZF-rep1 48349170 34032629 50 PE
ChIPseq-SET15-H3K14ac-EYFP-ZF-rep2 44200092 32570333 50 PE
ChIPseq-SET15-H3K14ac-fwa-rep1 44075233 34082299 50 PE
ChIPseq-SET15-H3K14ac-fwa-rep2 53313785 35890145 50 PE
ChIPseq-SET15-H3K27ac-EYFP-ZF-rep1 43602512 32254342 50 PE
ChIPseq-SET15-H3K27ac-EYFP-ZF-rep2 49632364 38520371 50 PE
ChIPseq-SET15-H3K27ac-fwa-rep1 50226011 39972700 50 PE
ChIPseq-SET15-H3K27ac-fwa-rep2 52110188 36735932 50 PE
ChIPseq-SET15-H3K9ac-EYFP-ZF-rep1_S41_L003 28632649 21285354 50 PE
ChIPseq-SET15-H3K9ac-EYFP-ZF-rep2 29897263 22824816 50 PE
ChIPseq-SET15-H3K9ac-fwa-rep1_S39_L003 29327321 23464787 50 PE
ChIPseq-SET15-H3K9ac-fwa-rep2 30198630 18853902 50 PE
ChIPseq-SET15-H4K16ac-EYFP-ZF-rep1_S52_L003 30508873 20630020 50 PE
ChIPseq-SET15-H4K16ac-EYFP-ZF-rep2 34912345 25075109 50 PE
ChIPseq-SET15-H4K16ac-fwa-rep1 37259605 27579510 50 PE

ChIPseq-SET3-H3K27ac-fwa1-rep1.bw
ChIPseq-SET3-H3K27ac-fwa2-rep1.bw
ChIPseq-SET3-H3K27ac-HD2A-ZF-rep1.bw
ChIPseq-SET3-H3K27ac-HD2B-ZF-rep1.bw
ChIPseq-SET3-H3K27ac-HD2C-ZF-rep1.bw
ChIPseq-SET3-H3K9ac-fwa1-rep1.bw
ChIPseq-SET3-H3K9ac-fwa2-rep1.bw
ChIPseq-SET3-H3K9ac-HD2A-ZF-rep1.bw
ChIPseq-SET3-H3K9ac-HD2B-ZF-rep1.bw
ChIPseq-SET3-H3K9ac-HD2C-ZF-rep1.bw
ChIPseq-SET3-H4K16ac-fwa1-rep1.bw
ChIPseq-SET3-H4K16ac-fwa2-rep1.bw
ChIPseq-SET3-H4K16ac-HD2A-ZF-rep1.bw
ChIPseq-SET3-H4K16ac-HD2B-ZF-rep1.bw
ChIPseq-SET3-H4K16ac-HD2C-ZF-rep1.bw
ChIPseq-SET4-H3-fwa1-rep2.bw
ChIPseq-SET4-H3-fwa2-rep2.bw
ChIPseq-SET4-H3-HD2A-ZF-rep2.bw
ChIPseq-SET4-H3-HD2B-ZF-rep2.bw
ChIPseq-SET4-H3-HD2C-ZF-rep2.bw
ChIPseq-SET4-H3K27ac-fwa1-rep2.bw
ChIPseq-SET4-H3K27ac-fwa2-rep2.bw
ChIPseq-SET4-H3K27ac-HD2A-ZF-rep2.bw
ChIPseq-SET4-H3K27ac-HD2B-ZF-rep2.bw
ChIPseq-SET4-H3K27ac-HD2C-ZF-rep2.bw
ChIPseq-SET5-H3-fwa-rep2.bw
ChIPseq-SET5-H3-HD2A-ZF-rep2.bw
ChIPseq-SET5-H3-HD2B-ZF-rep2.bw
ChIPseq-SET5-H3-HD2C-ZF-rep2.bw
ChIPseq-SET5-H4K16ac-fwa-rep2.bw
ChIPseq-SET5-H4K16ac-HD2A-ZF-rep2.bw
ChIPseq-SET5-H4K16ac-HD2B-ZF-rep2.bw
ChIPseq-SET5-H4K16ac-HD2C-ZF-rep2.bw
ChIPseq-SET6-H3-fwa-rep2.bw
ChIPseq-SET6-H3-HD2A-ZF-rep2.bw
ChIPseq-SET6-H3K9ac-fwa-rep2.bw
ChIPseq-SET6-H3K9ac-HD2A-ZF-rep2.bw
ChIPseq-SET7-H3-fwa-rep1.bw
ChIPseq-SET7-H3-fwa-rep2_S2_L004.bw
ChIPseq-SET7-H3-HDA6-ZF-rep1_S5_L004.bw
ChIPseq-SET7-H3-HDA6-ZF-rep2_S6_L004.bw
ChIPseq-SET7-H3-MSI1-ZF-rep1_S3_L004.bw
ChIPseq-SET7-H3-MSI1-ZF-rep2_S4_L004.bw
ChIPseq-SET7-H3-MSI1-ZF-rep2_S4_L004.bw
ChIPseq-SET7-H3K14ac-fwa-rep1_S13_L004.bw
ChIPseq-SET7-H3K14ac-fwa-rep2_S14_L004.bw
ChIPseq-SET7-H3K14ac-HDA6-ZF-rep1_S17_L004.bw
ChIPseq-SET7-H3K14ac-HDA6-ZF-rep2_S18_L004.bw
ChIPseq-SET7-H3K14ac-MSI1-ZF-rep1_S15_L004.bw
ChIPseq-SET7-H3K14ac-MSI1-ZF-rep2_S16_L004.bw
ChIPseq-SET7-H3K9ac-fwa-rep1_S7_L004.bw
ChIPseq-SET7-H3K9ac-fwa-rep2_S8_L004.bw
ChIPseq-SET7-H3K9ac-HDA6-ZF-rep1_S11_L004.bw
ChIPseq-SET7-H3K9ac-HDA6-ZF-rep2_S12_L004.bw
ChIPseq-SET7-H3K9ac-MSI1-ZF-rep1_S9_L004.bw
ChIPseq-SET7-H3K9ac-MSI1-ZF-rep2_S10_L004.bw
ChIPseq-SET8-H3-ELF7-ZF-rep1.bw
ChIPseq-SET8-H3-fwa-rep1.bw
ChIPseq-SET8-H3-HD2A-ZF-rep1.bw
ChIPseq-SET8-PolII-ELF7-ZF-rep1.bw
ChIPseq-SET8-PolII-fwa-rep1.bw
ChIPseq-SET8-PolII-HD2A-ZF-rep1.bw
ChIPseq-SET9-H3-ELF7-ZF-rep2.bw
ChIPseq-SET9-H3-fwa-rep2.bw
ChIPseq-SET9-H3-HD2A-ZF-rep2.bw
ChIPseq-SET9-PolII-ELF7-ZF-rep2.bw
ChIPseq-SET9-PolII-fwa-rep2.bw
ChIPseq-SET9-PolII-HD2A-ZF-rep2.bw

Genome browser session (e.g. UCSC)

Available at GEO

ChIPseq-SET15-H4K16ac-fwa-rep2_S51_L003 26181976 17141717 50 PE

ChIPseq-SET16-FLAG-Col-0-rep1 22729523 20665012 50 PE
ChIPseq-SET16-FLAG-ELF7-rep1 39467014 37902583 50 PE
ChIPseq-SET16-FLAG-fwa 23220319 21012927 50 PE
ChIPseq-SET16-FLAG-TRB1-ZF 33811422 27025458 50 PE
ChIPseq-SET17-FLAG-Col-0-rep2 37514884 31311145 50 PE
ChIPseq-SET17-FLAG-ELF7-rep2 23428561 11957299 50 PE
ChIPseq-SET2-H3-ELF7-ZF-rep2 22838411 21289938 50 PE
ChIPseq-SET2-H3-fwa-rep2 22025700 20443743 50 PE
ChIPseq-SET2-H3-JMJ14-ZF-rep2 22725481 21144808 50 PE
ChIPseq-SET2-H3-LHP1-ZF-rep2 24485900 22758104 50 PE
ChIPseq-SET2-H3-MSI1-ZF-rep2 24569985 22975528 50 PE
ChIPseq-SET2-H3K27me3-ELF7-ZF-rep2 10475493 9425074 50 PE
ChIPseq-SET2-H3K27me3-fwa-rep2 12199070 11197483 50 PE
ChIPseq-SET2-H3K27me3-JMJ14-ZF-rep2 18540799 16428979 50 PE
ChIPseq-SET2-H3K27me3-LHP1-ZF-rep2 11286871 10295854 50 PE
ChIPseq-SET2-H3K27me3-MSI1-ZF-rep2 11056992 10138340 50 PE
ChIPseq-SET2-H3K4me3-ELF7-ZF-rep2 15936122 14500824 50 PE
ChIPseq-SET2-H3K4me3-fwa-rep2 17925369 16471918 50 PE
ChIPseq-SET2-H3K4me3-JMJ14-ZF-rep2 18405310 16961254 50 PE
ChIPseq-SET20-H3K36me2-ELF7-ZF-rep2 23247296 21276627 50 PE
ChIPseq-SET20-H3K36me2-fwa-rep2 18882469 17293941 50 PE
ChIPseq-SET20-H3K36me3-ELF7-ZF-rep2 25033667 23307872 50 PE
ChIPseq-SET20-H3K36me3-fwa-rep2 22173784 20981888 50 PE
ChIPseq-SET3-H3-fwa1-rep1 27580626 25957889 50 PE
ChIPseq-SET3-H3-fwa2-rep1 25749655 24236137 50 PE
ChIPseq-SET3-H3-HD2A-ZF-rep1 24580547 23103541 50 PE
ChIPseq-SET3-H3-HD2B-ZF-rep1 22916104 21599960 50 PE
ChIPseq-SET3-H3-HD2C-ZF-rep1 28289837 26824617 50 PE
ChIPseq-SET3-H3K27ac-fwa1-rep1 19966907 18757693 50 PE
ChIPseq-SET3-H3K27ac-fwa2-rep1 26703192 25071995 50 PE
ChIPseq-SET3-H3K27ac-HD2A-ZF-rep1 24591618 23019497 50 PE
ChIPseq-SET3-H3K27ac-HD2B-ZF-rep1 24342292 22791146 50 PE
ChIPseq-SET3-H3K27ac-HD2C_ZF-rep1 22924643 21556266 50 PE
ChIPseq-SET3-H3K9ac-fwa1-rep1_L001 15046194 13660906 50 PE
ChIPseq-SET3-H3K9ac-fwa2-rep1 14605579 13144964 50 PE
ChIPseq-SET3-H3K9ac-HD2A_ZF-rep1 15883814 14712197 50 PE
ChIPseq-SET3-H3K9ac-HD2B_ZF-rep1 19020238 17283977 50 PE
ChIPseq-SET3-H3K9ac-HD2C_ZF-rep1 16076147 14840973 50 PE
ChIPseq-SET3-H4K16ac-fwa1-rep1 17943856 15673765 50 PE
ChIPseq-SET3-H4K16ac-fwa2-rep1 17456211 15757486 50 PE
ChIPseq-SET3-H4K16ac-HD2A_ZF-rep1 17988496 14661974 50 PE
ChIPseq-SET3-H4K16ac-HD2B_ZF-rep1 20507006 18523393 50 PE
ChIPseq-SET3-H4K16ac-HD2C_ZF-rep1 20775891 19095773 50 PE
ChIPseq-SET4-H3-fwa1-rep2 19507713 16172889 50 PE
ChIPseq-SET4-H3-fwa2-rep2 18656208 16321569 50 PE
ChIPseq-SET4-H3-HD2A-ZF-rep2 18335079 16055593 50 PE
ChIPseq-SET4-H3-HD2B-ZF-rep2 15705178 13301568 50 PE
ChIPseq-SET4-H3-HD2C-ZF-rep2 17052885 15273277 50 PE
ChIPseq-SET4-H3K27ac-fwa1-rep2 17882609 14477275 50 PE
ChIPseq-SET4-H3K27ac-fwa2-rep2-L2_S14_L001 20749781 17409462 50 PE
ChIPseq-SET4-H3K27ac-HD2A-ZF-rep2 17540431 14993438 50 PE
ChIPseq-SET4-H3K27ac-HD2B-ZF-rep2 18024767 14509791 50 PE
ChIPseq-SET4-H3K27ac-HD2C-ZF-rep2 20821423 18159479 50 PE
ChIPseq-SET6-H3-fwa-rep2 22655329 20188042 50 PE
ChIPseq-SET6-H3-HD2A-ZF-rep2 22803021 19714849 50 PE
ChIPseq-SET6-H3K9ac-fwa-rep2 15554264 13730331 50 PE
ChIPseq-SET6-H3K9ac-HD2A-ZF-rep2 15226346 12896220 50 PE
ChIPseq-SET7-H3-fwa-rep1 76530470 59371691 50 PE
ChIPseq-SET7-H3-fwa-rep2 73226486 56989879 50 PE
ChIPseq-SET7-H3-HDA6-ZF-rep1 71569269 54020339 50 PE
ChIPseq-SET7-H3-HDA6-ZF-rep2 83928105 62098263 50 PE
ChIPseq-SET7-H3-MSI1-ZF-rep1 69634348 51567135 50 PE
ChIPseq-SET7-H3-MSI1-ZF-rep2 70831417 54131485 50 PE
ChIPseq-SET7-H3K14ac-fwa-rep1 58025194 37692004 50 PE
ChIPseq-SET7-H3K14ac-fwa-rep2 57747168 38277608 50 PE
ChIPseq-SET7-H3K14ac-HDA6-ZF-rep1 54746582 35427315 50 PE
ChIPseq-SET7-H3K14ac-HDA6-ZF-rep2 52922135 33555963 50 PE
ChIPseq-SET7-H3K14ac-MSI1-ZF-rep1 51613891 32836307 50 PE
ChIPseq-SET7-H3K14ac-MSI1-ZF-rep2 64916246 44548004 50 PE
ChIPseq-SET7-H3K9ac-fwa-rep1 46702785 32031581 50 PE
ChIPseq-SET7-H3K9ac-fwa-rep2 54680530 38515159 50 PE
ChIPseq-SET7-H3K9ac-HDA6-ZF-rep1 48609483 33672170 50 PE
ChIPseq-SET7-H3K9ac-HDA6-ZF-rep2 48565834 31568178 50 PE
ChIPseq-SET7-H3K9ac-MSI1-ZF-rep1 64272604 41450531 50 PE
ChIPseq-SET7-H3K9ac-MSI1-ZF-rep2 56971803 37847234 50 PE
ChIPseq-SET8-H3-ELF7-ZF-rep1_S43_L003 28762659 25652952 50 PE

ChIPseq-SET8-H3-fwa-rep1 33391447 30985333 50 PE
ChIPseq-SET8-H3-HD2A-ZF-rep1 28408697 26352421 50 PE
ChIPseq-SET8-PolII-ELF7-ZF-rep1_S54_L003 22931326 19386760 50 PE
ChIPseq-SET8-PolII-fwa-rep1 21615655 17293168 50 PE
ChIPseq-SET8-PolII-HD2A-ZF-rep1 26388420 22619253 50 PE
ChIPseq-SET9-H3-ELF7-ZF-rep2_S2_L001 26619337 24867631 50 PE
ChIPseq-SET9-H3-fwa-rep2 26871683 25416722 50 PE
ChIPseq-SET9-H3-HD2A-ZF-rep2 23227253 21677325 50 PE
ChIPseq-SET9-PolII-ELF7-ZF-rep2 8780828 7854858 50 PE
ChIPseq-SET9-PolII-fwa-rep2 9302660 8406014 50 PE
ChIPseq-SET9-PolII-HD2A-ZF-rep2 7529608 6408422 50 PE

Antibodies

Anti-H3K27me3 (Millipore Sigma)
Anti-H3 (Abcam)
Anti-H3K4me3 (Millipore Sigma)
Anti-H3K27ac (Abcam)
Anti-H3K9ac (Abcam)
Anti-H4K16ac (Abcam)
Anti-H3K36me2 (Abcam)
Anti-H3K14ac (Abcam)
anti-Pol II Ser 5 (Abcam)
anti-FLAG M2 (Sigma)
Anti-H3K36me3 (Abcam)

Peak calling parameters

MACS2: '-f BAM -g 1.3e+8 -q 0.05 --extsize 147'

Data quality

All identified peaks in the study were called with a qval threshold of 0.01 (FDR 1%).

Software

Bowtie (v1.1.2),
Samtools (v1.9)
MACS2 (v2.1.1)
ChIPseeker
deeptools (v2.5.1).
bedtools (v2.26.0)
