# Supplementary figures and images for: A gene silencing screen uncovers diverse tools for targeted gene repression in Arabidopsis
Source: Nat Plants. 2023 Mar 6;9(3):460–72. doi: 10.1038/s41477-023-01362-8 (PMC10027610; doi:10.1038/s41477-023-01362-8)

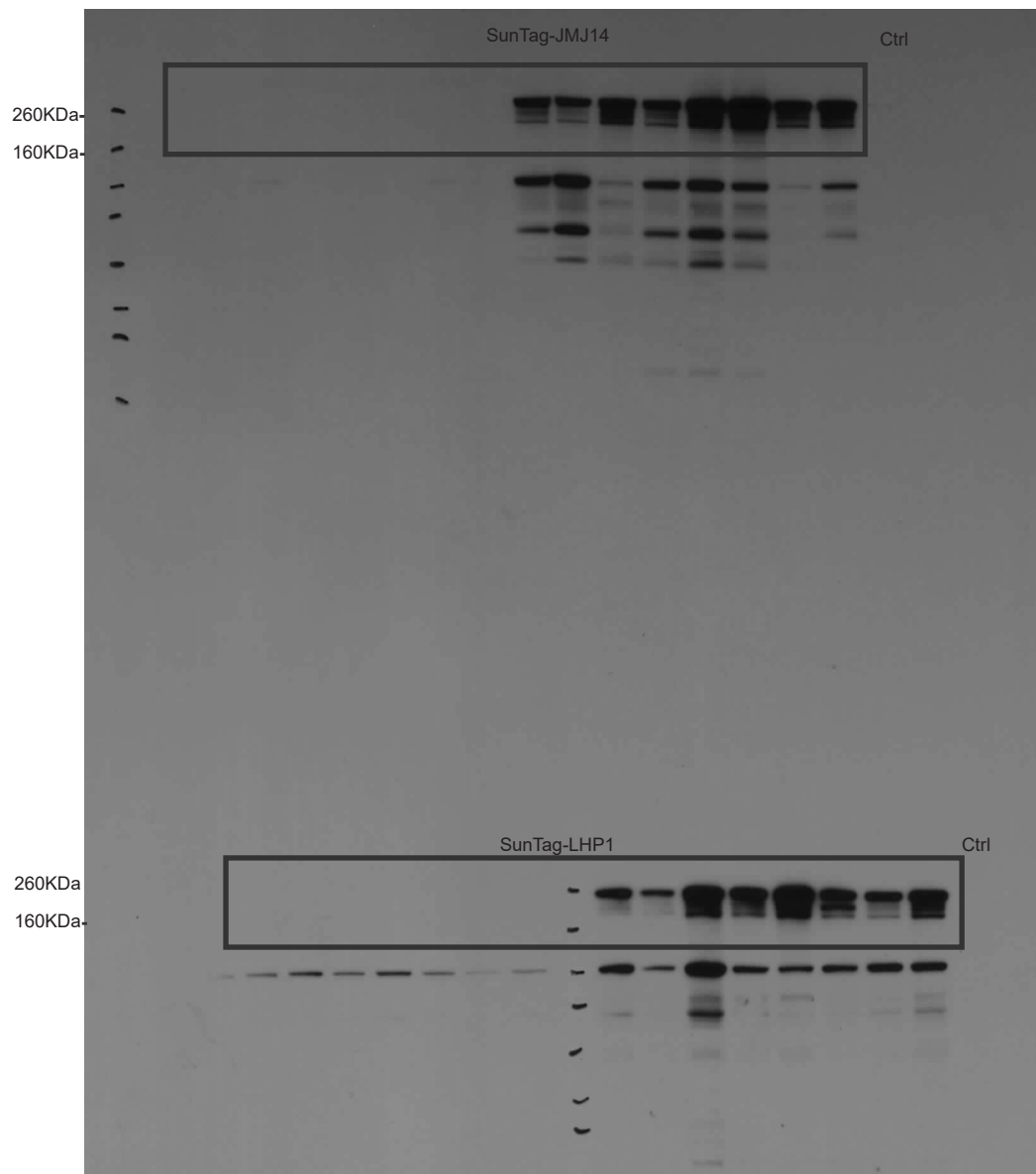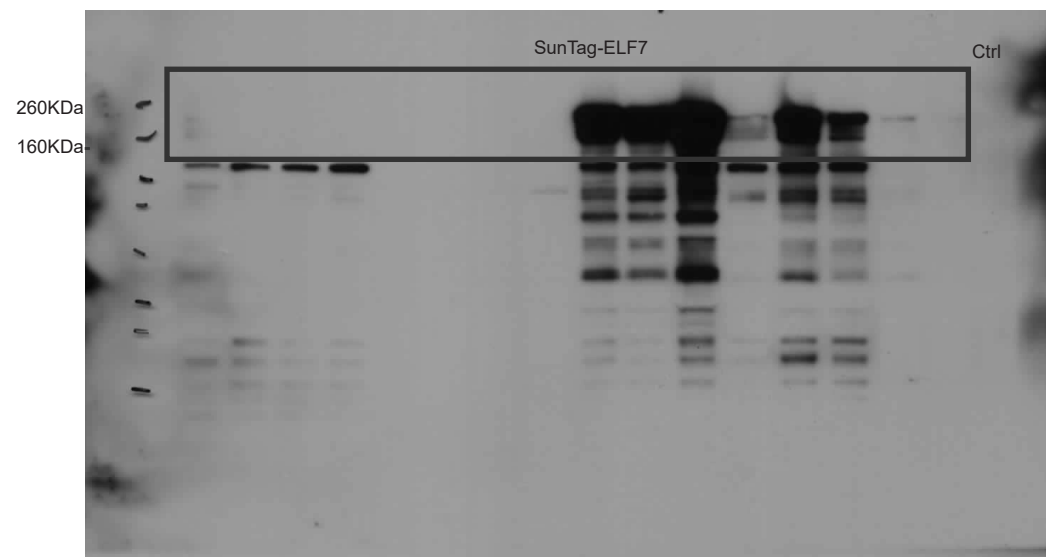

Supplement: Source Data Fig. 2 — Unprocessed western blots of Fig. 2c. [file 41477_2023_1362_MOESM4_ESM.pdf]

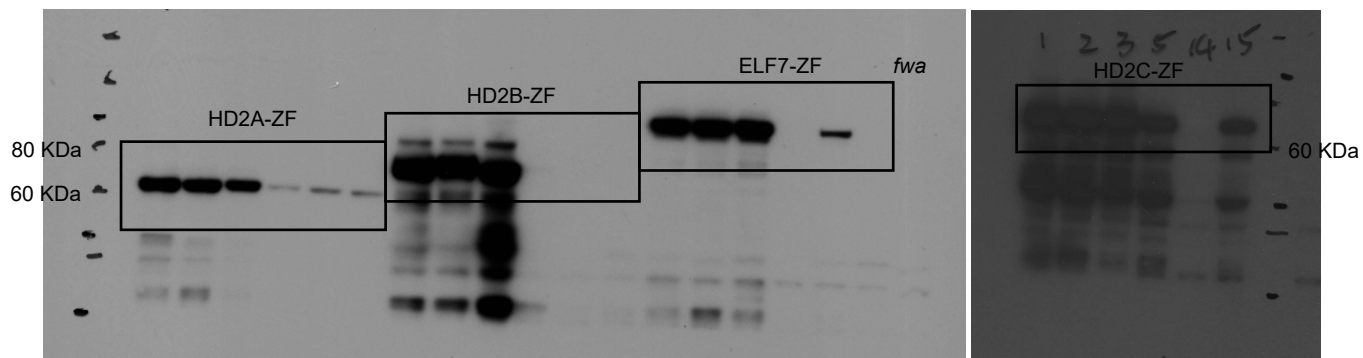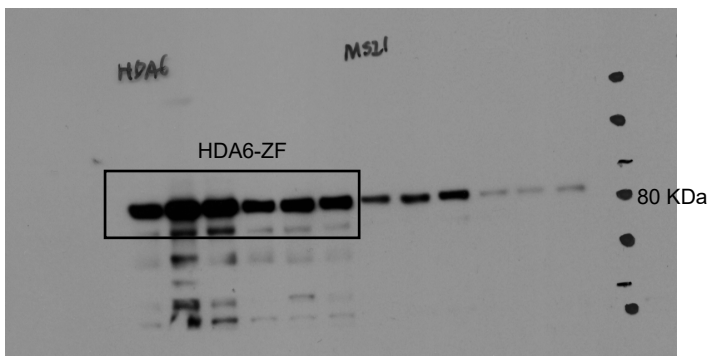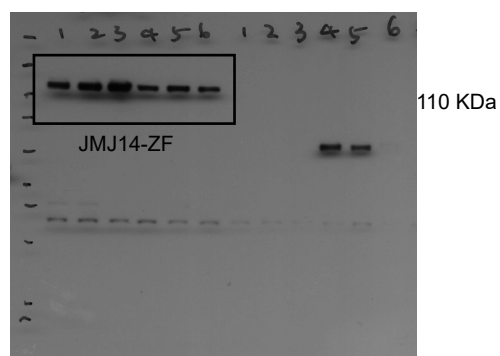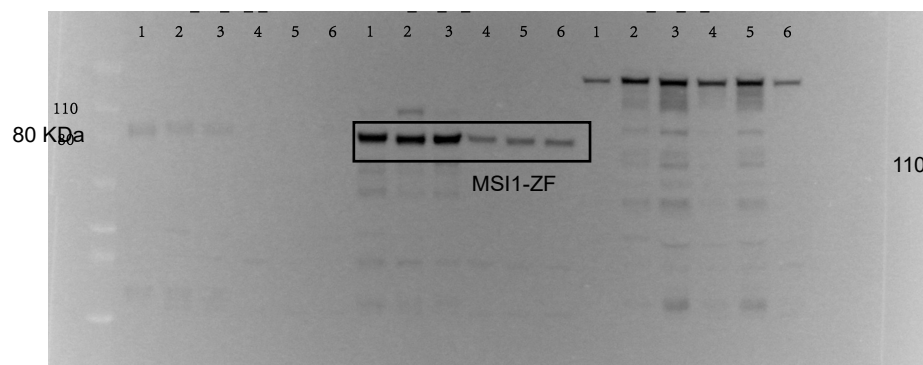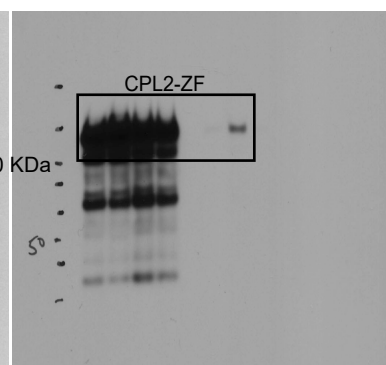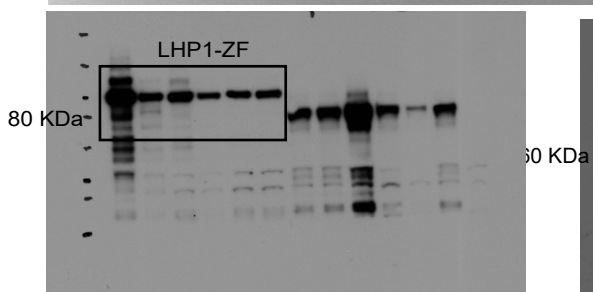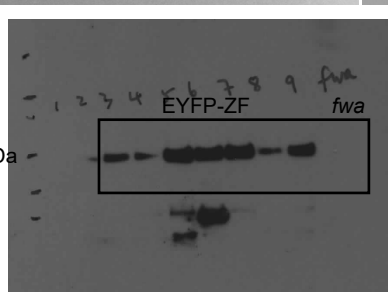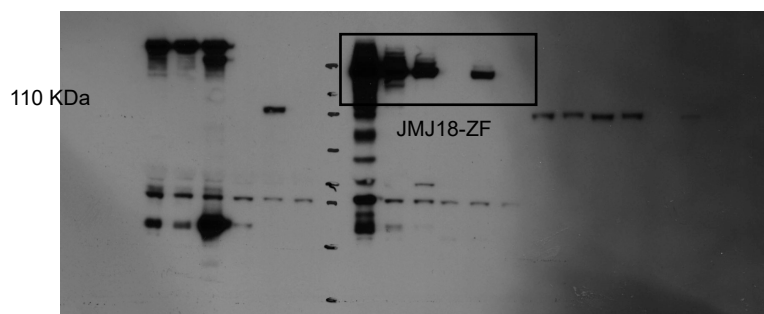

Supplement: Source Data Extended Data Fig./Table 1 — Unprocessed western blots of Extended Data Fig. 1a. [file 41477_2023_1362_MOESM5_ESM.pdf]
